# Supplementary figures and images for: Unique ecology of co-occurring functionally and phylogenetically undescribed species in the infant oral microbiome
Source: PLoS Comput Biol. 2026 Mar 5;22(3):e1013185. doi: 10.1371/journal.pcbi.1013185 (PMC13004514; doi:10.1371/journal.pcbi.1013185)

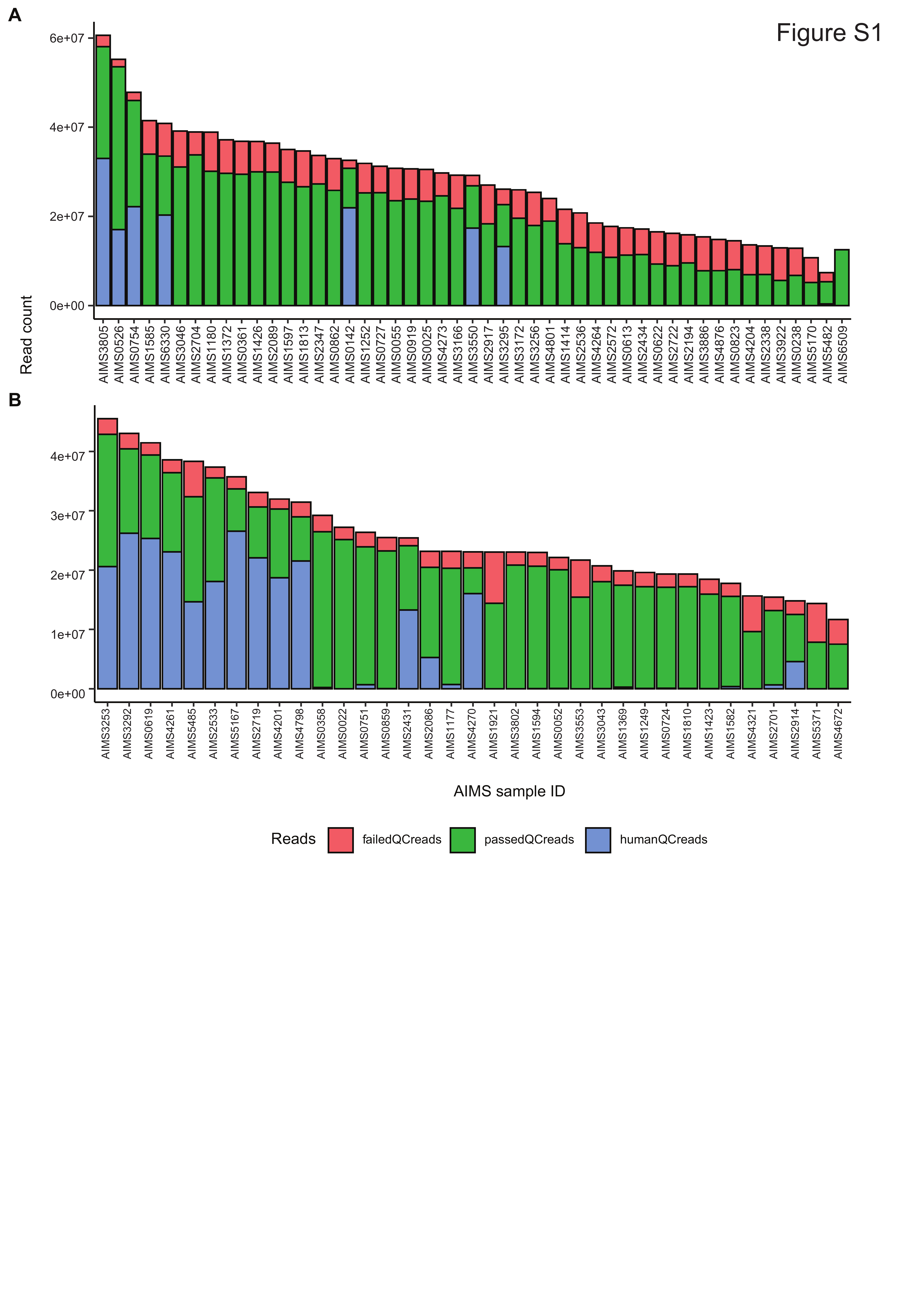

Supplement: S1 Fig — For each bar, colors indicate the proportion of reads from A) tongue and B) tooth biofilm samples that passed (green) and failed (red) quality control as well as reads mapping to human DNA (blue). Failed and human reads were subsequently filtered out the dataset. (TIF) [file pcbi.1013185.s001.tif]

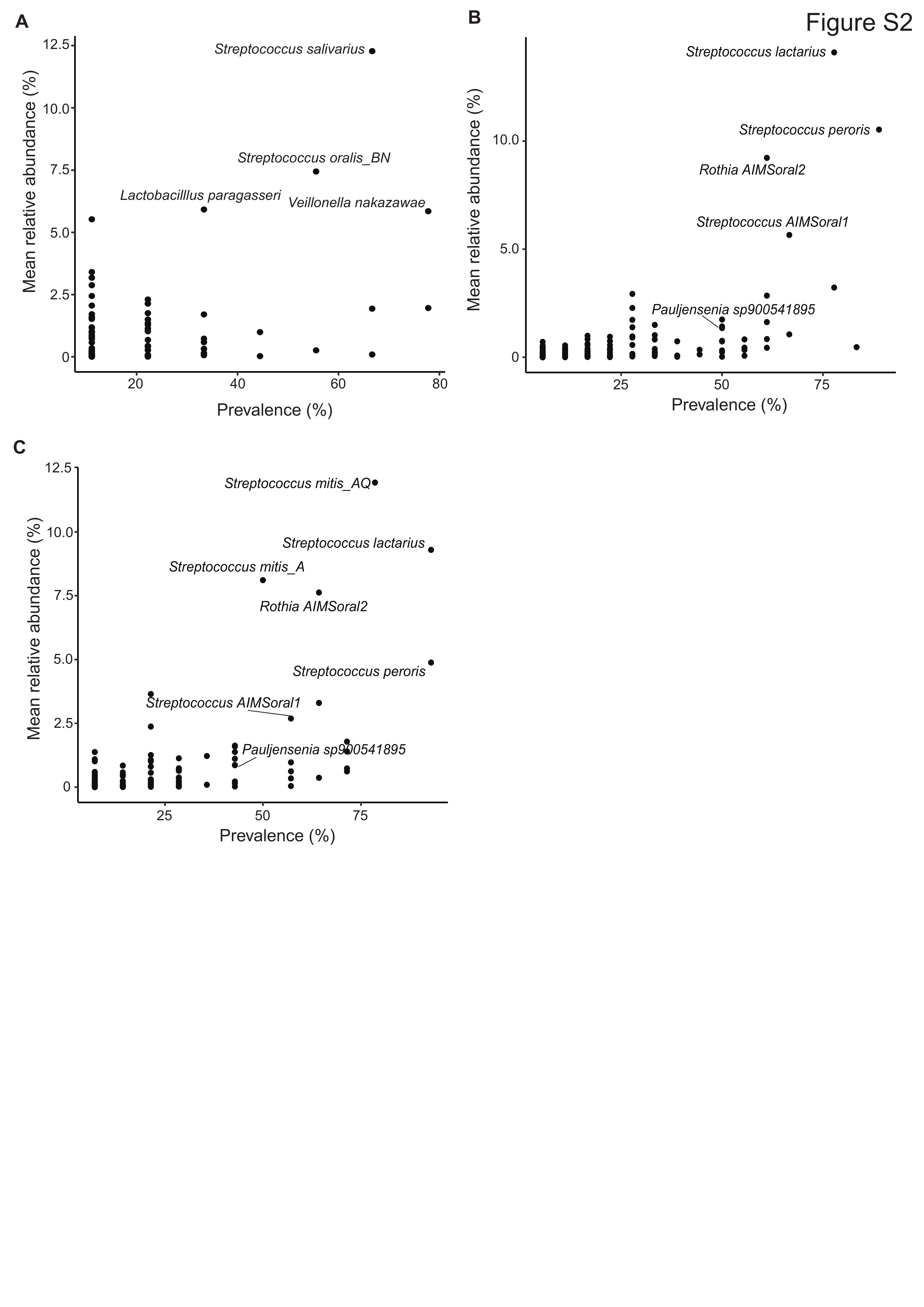

Supplement: S2 Fig — Mean relative abundance versus prevalence for bacterial species detected in infant oral samples. Labeled species represent the most abundant and prevalent taxa at each timepoint. (A) 1-month tongue (n = 9). (B) 6-month tongue (n = 18). (C) 6-month tooth (n = 14). Streptococcus AIMSoral1 and Rothia AIMSoral2 are among the most abundant and prevalent species at 6 months, along with other infant-associated streptococci and Pauljensenia sp900541895. (TIF) [file pcbi.1013185.s002.tif]

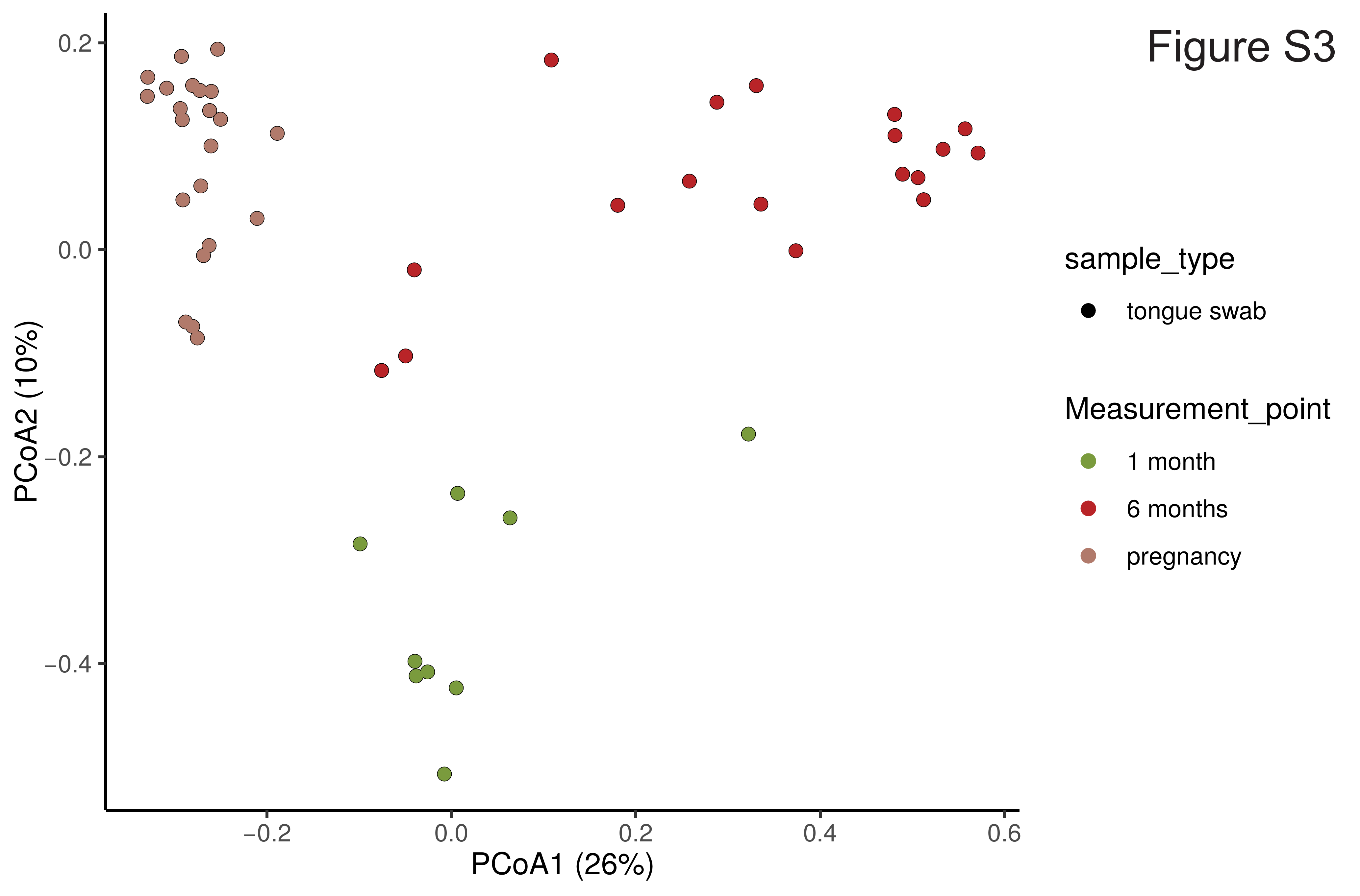

Supplement: S3 Fig — (TIF) [file pcbi.1013185.s003.tif]

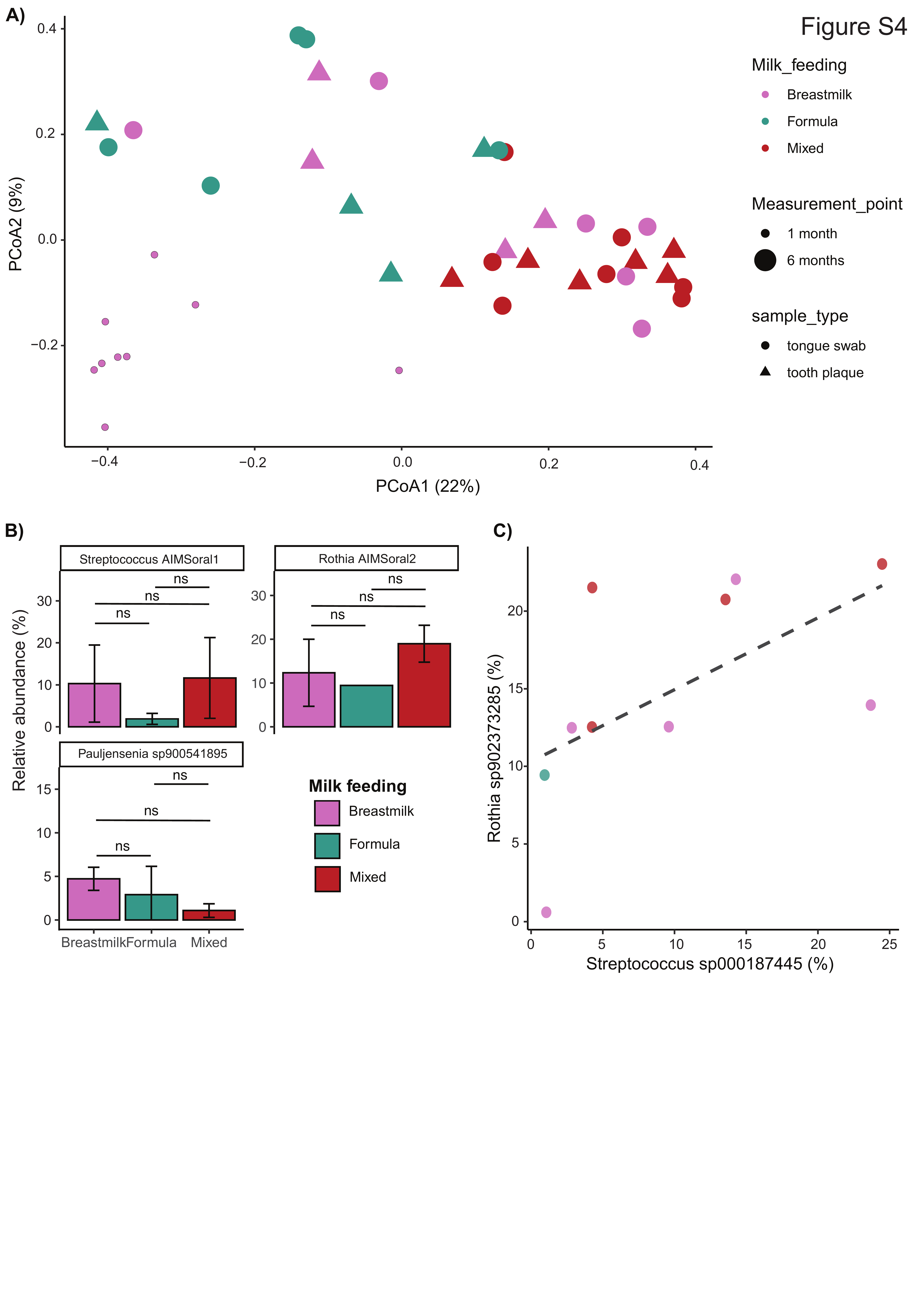

Supplement: S4 Fig — A) PCoA of tongue dorsum (circles) and dental plaque (triangles) microbiomes from AIMS infants (1 month = small; 6 months = large). For each sample, the corresponding milk feeding type is reported (breastmilk = pink, mixed = red,formula = green). PERMANOVA analyses showed no significant clustering based on milk feeding within the 6 months samples (adonis: R2 = 0.07, F = 1.10, p = 0.32). B) Relative abundances of Streptococcus AIMSoral1, Rothia AIMSoral2 and Pauljensenia sp900541895 found in infants breast-, formula- and mixed milk-feeding. Significance among groups was tested for each species by pairwise Wilcoxon’s signed rank tests. No significant differences were detected. C) Correlation plot of Streptococcus AIMSoral1 and Rothia AIMSoral2 showed no association between their abundance and milk-feeding type. (TIF) [file pcbi.1013185.s004.tif]

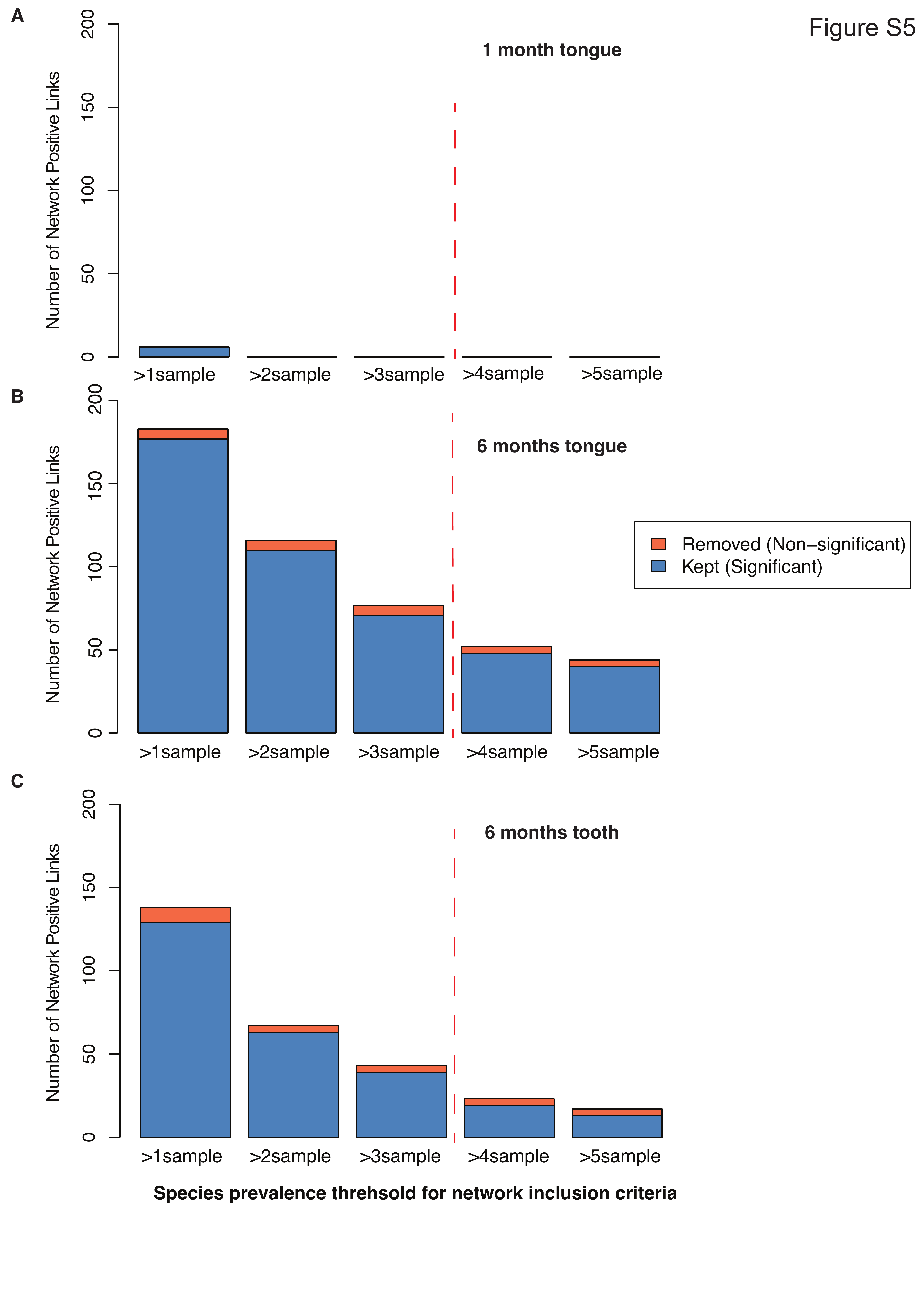

Supplement: S5 Fig — Co-abundance networks were constructed using SPIEC-EASI for species present above different prevalence thresholds (>1 to >5 samples) and corroborated using Spearman’s rank correlation. Bars show the number of positive network interactions (edges/links) identified by SPIEC-EASI at each threshold. Blue segments represent edges supported by significant Spearman correlations (p ≤ 0.05; “kept”), while orange segments represent edges lacking Spearman significance (“removed”). The red dashed line indicates the selected threshold (>4 samples), chosen to minimize discordance between SPIEC-EASI edges and Spearman correlation significance while maintaining network complexity. A) 1-month tongue samples (n = 9). B) 6-month tongue samples (n = 18). C) 6-month tooth samples (n = 14). At 1 month, no significant co-abundance relationships were detected regardless of threshold. (TIF) [file pcbi.1013185.s005.tif]

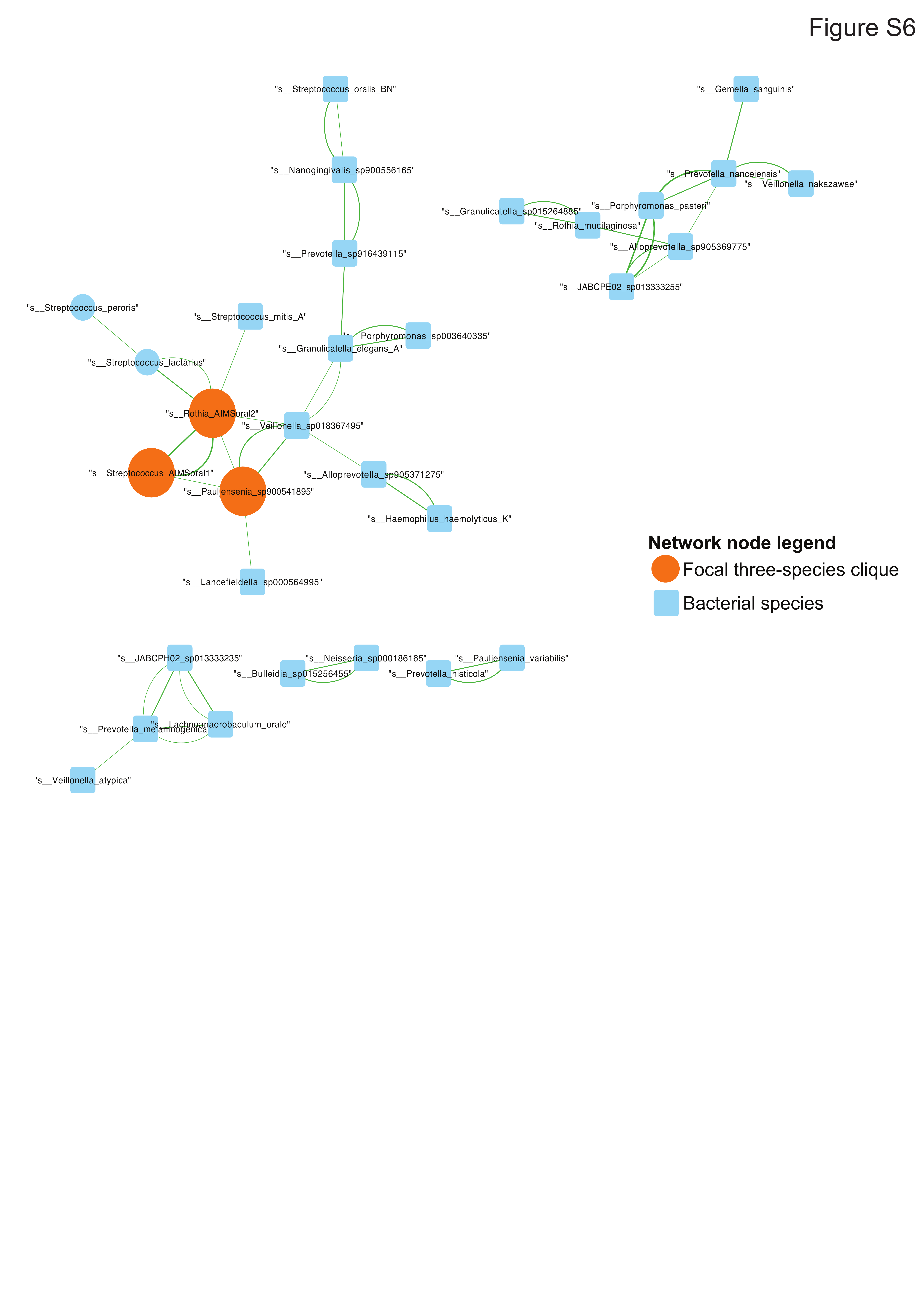

Supplement: S6 Fig — SPIEC-EASI network from 6-month infant tongue samples. Network inference was carried out only on species present in at least 5 samples. Only network links supported by significant Spearman’s rank correlations are shown. Orange nodes: undescribed Streptococcus AIMSoral1, Rothia AIMSoral2 and co-occurring Pauljensenia sp900541895 selected for metabolic modeling. Blue nodes: other bacterial species. Edges width indicates the strength of the association based on SPIEC-EASI covariance. (TIF) [file pcbi.1013185.s006.tif]

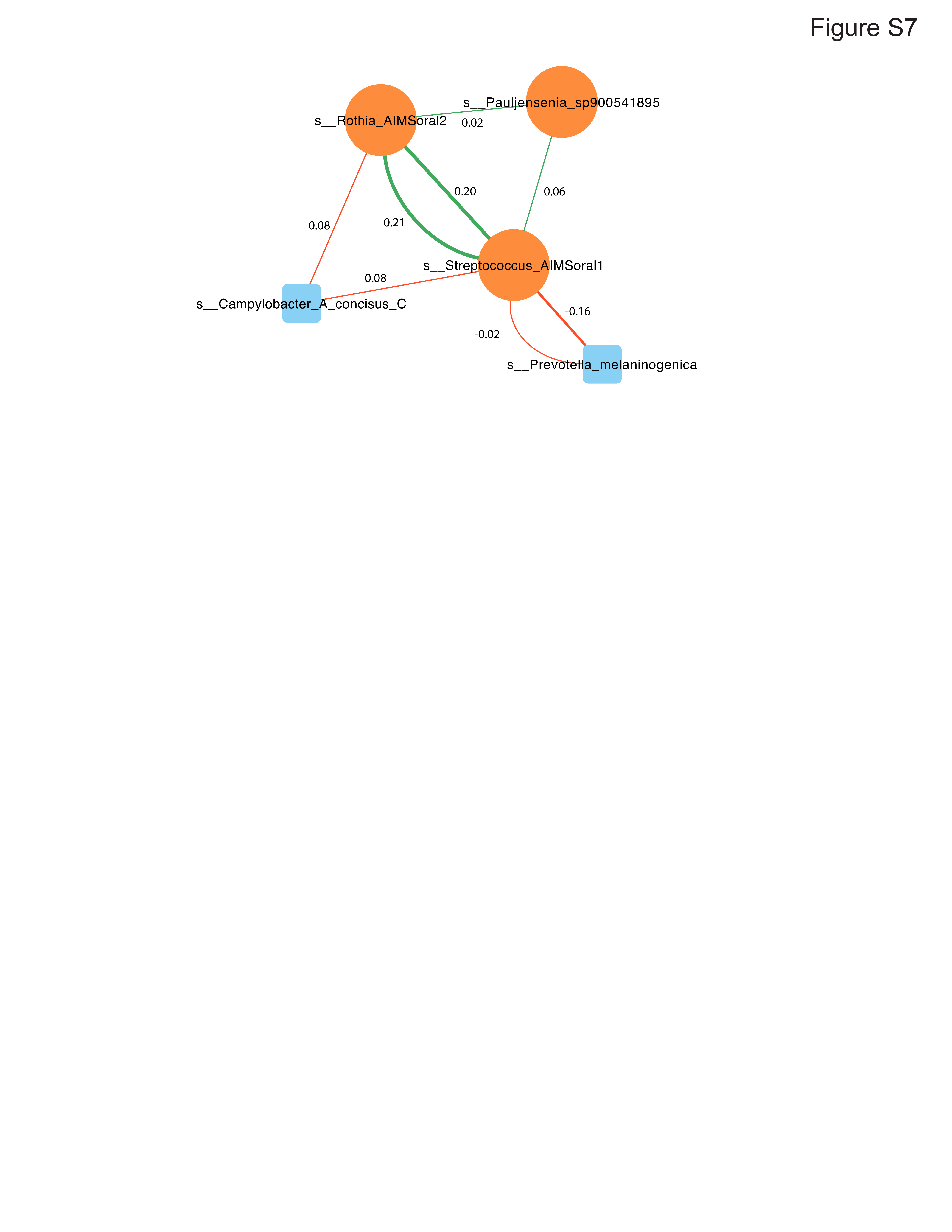

Supplement: S7 Fig — Subset of the SPIEC-EASI network from 6-month infant tongue samples showing direct interactions of focal species. Orange nodes: undescribed Streptococcus AIMSoral1, Rothia AIMSoral2 and co-occurring Pauljensenia sp900541895 selected for metabolic modeling. Blue nodes: species exhibiting negative co-abundance associations with focal species. Green edges indicate positive associations; red edges indicate negative associations. Edge labels show SPIEC-EASI covariance values. (TIF) [file pcbi.1013185.s007.tif]

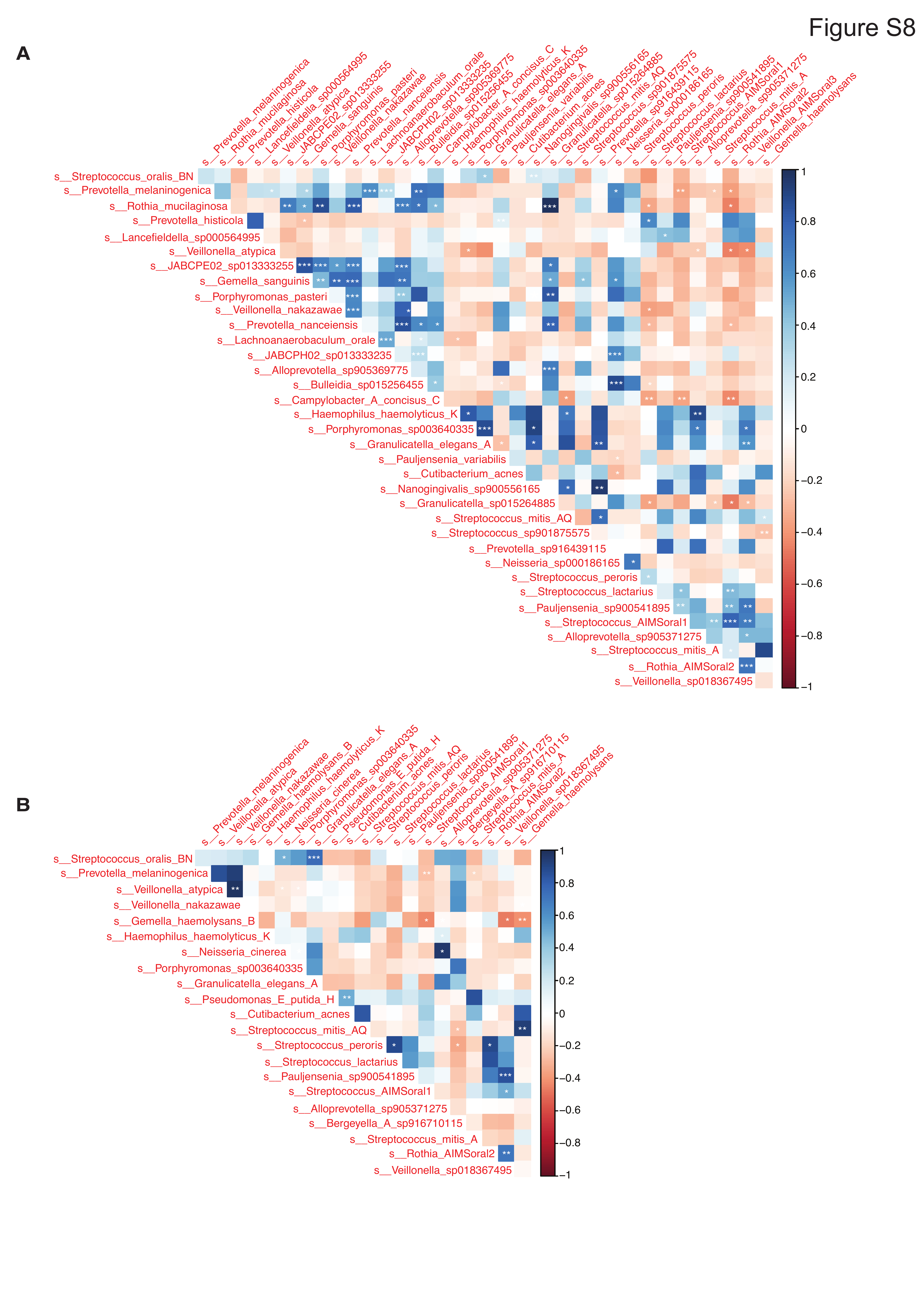

Supplement: S8 Fig — Correlations between oral species in A) the tongue biofilm and B) dental biofilm of 6-month-old AIMS infants are colored in blue (positive) or red (negative). Significance is indicated by asterisks: *p ≤ 0.05, ** ≤ 0.01, *** ≤ 0.001, **** ≤ 0.0001. Spearman’s rank correlations were carried out only among species present in at least 5 samples per group. (TIF) [file pcbi.1013185.s008.tif]

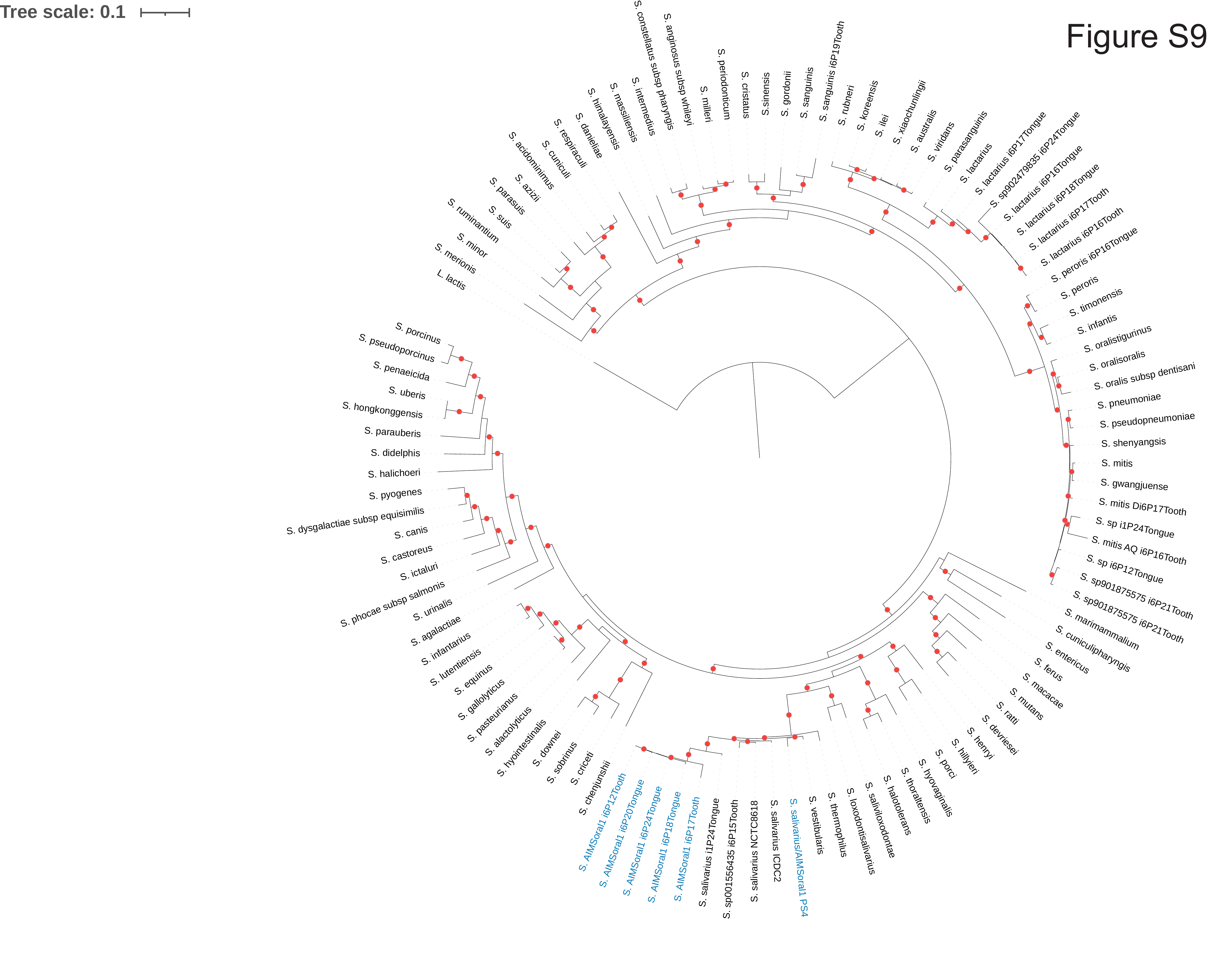

Supplement: S9 Fig — Phylogenomic analysis of 110 Streptococcus genomes representing 89 species, including 89 reference genomes and 21 metagenome-assembled genomes (MAGs). Lactococcus lactis was included as an outgroup. Red circles indicate bootstrap values≥0.9. (TIF) [file pcbi.1013185.s009.tif]

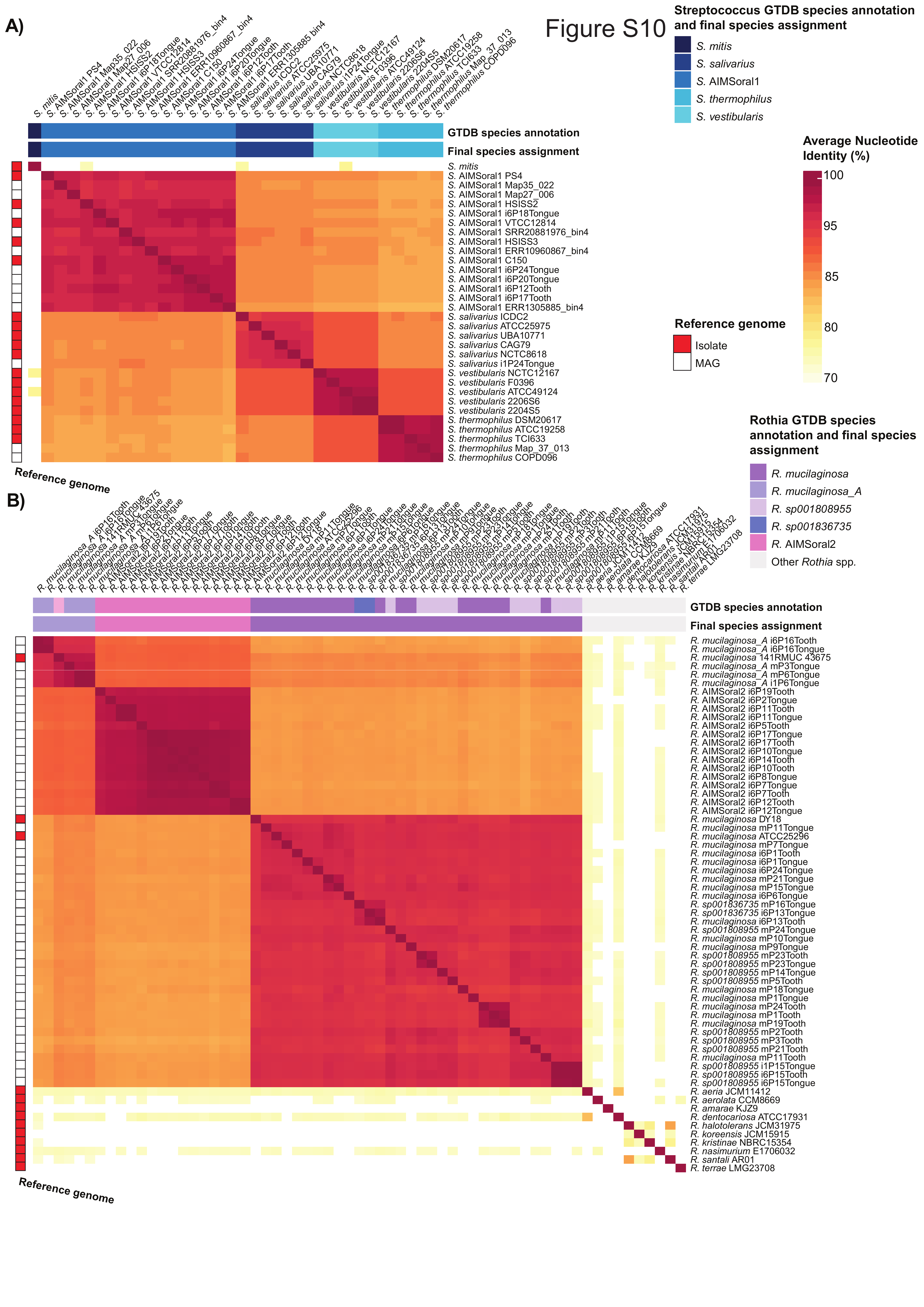

Supplement: S10 Fig — Pairwise ANI comparisons between A) Streptococcus (salivarius group) and B) Rothia MAGs (white) and reference genomes (red). For each genome, the GTDB/AIMSoral and final species assignments are provided. (TIF) [file pcbi.1013185.s010.tif]

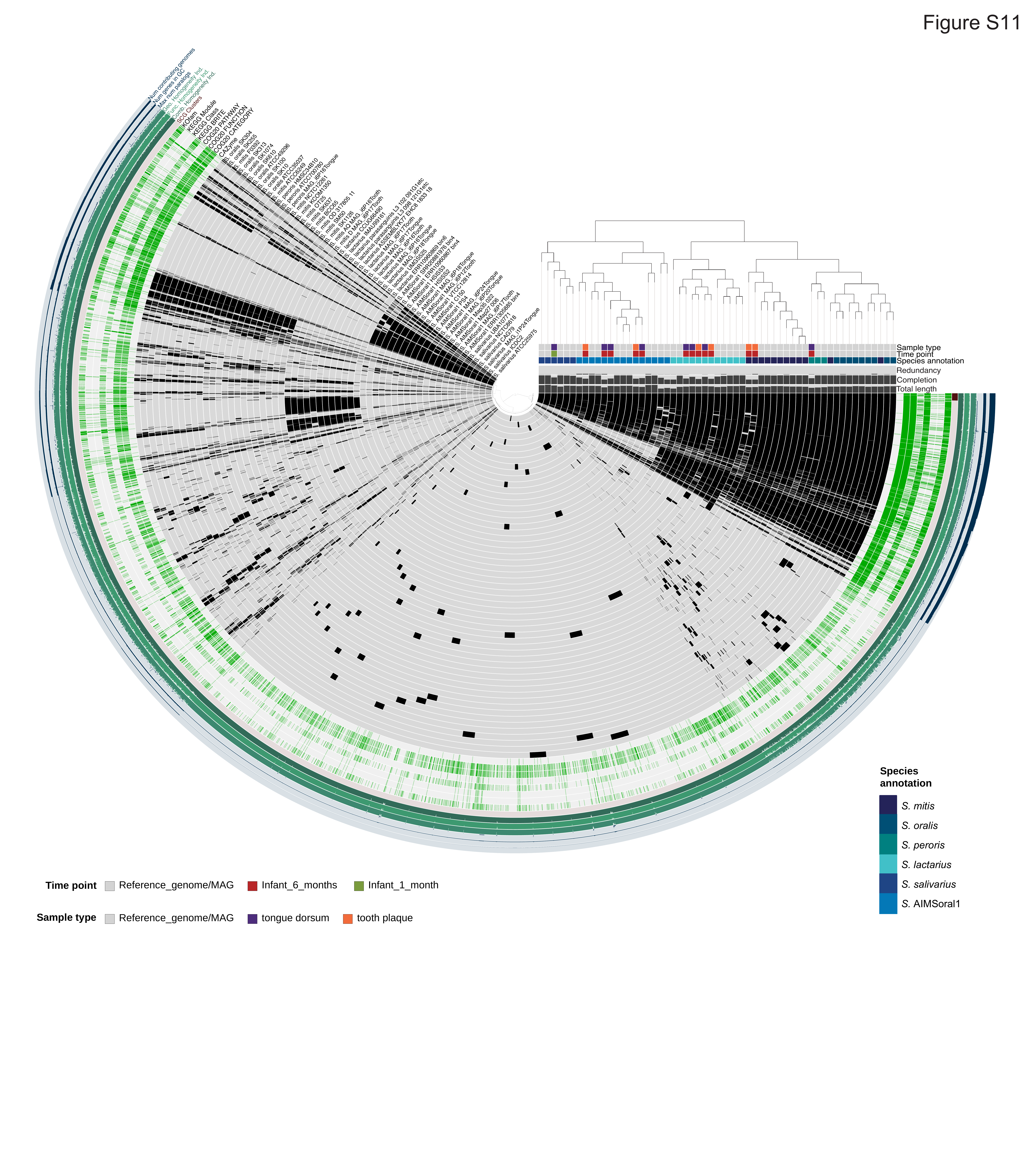

Supplement: S11 Fig — The figure shows the pangenomic characteristics of infant-associated Streptococcus species sorted by gene presence/absence. The pangenome includes MAGs reconstructed from AIMS oral samples (sampling location: tongue dorsum = purple, tooth plaque = orange) collected at different timepoints (infant 1 month = green, infant 6 months = red). No high- and medium-quality MAGs from infant-associated species were retrieved from maternal oral samples. Reference genomes are given in grey. Species annotations as well as bars showing genome/MAG total length, completion and redundancy are shown for each strain. For each gene cluster, metrics such as number of genomes where a gene cluster is present (’num of contributing genomes) and various functional annotations are shown. (TIF) [file pcbi.1013185.s011.tif]

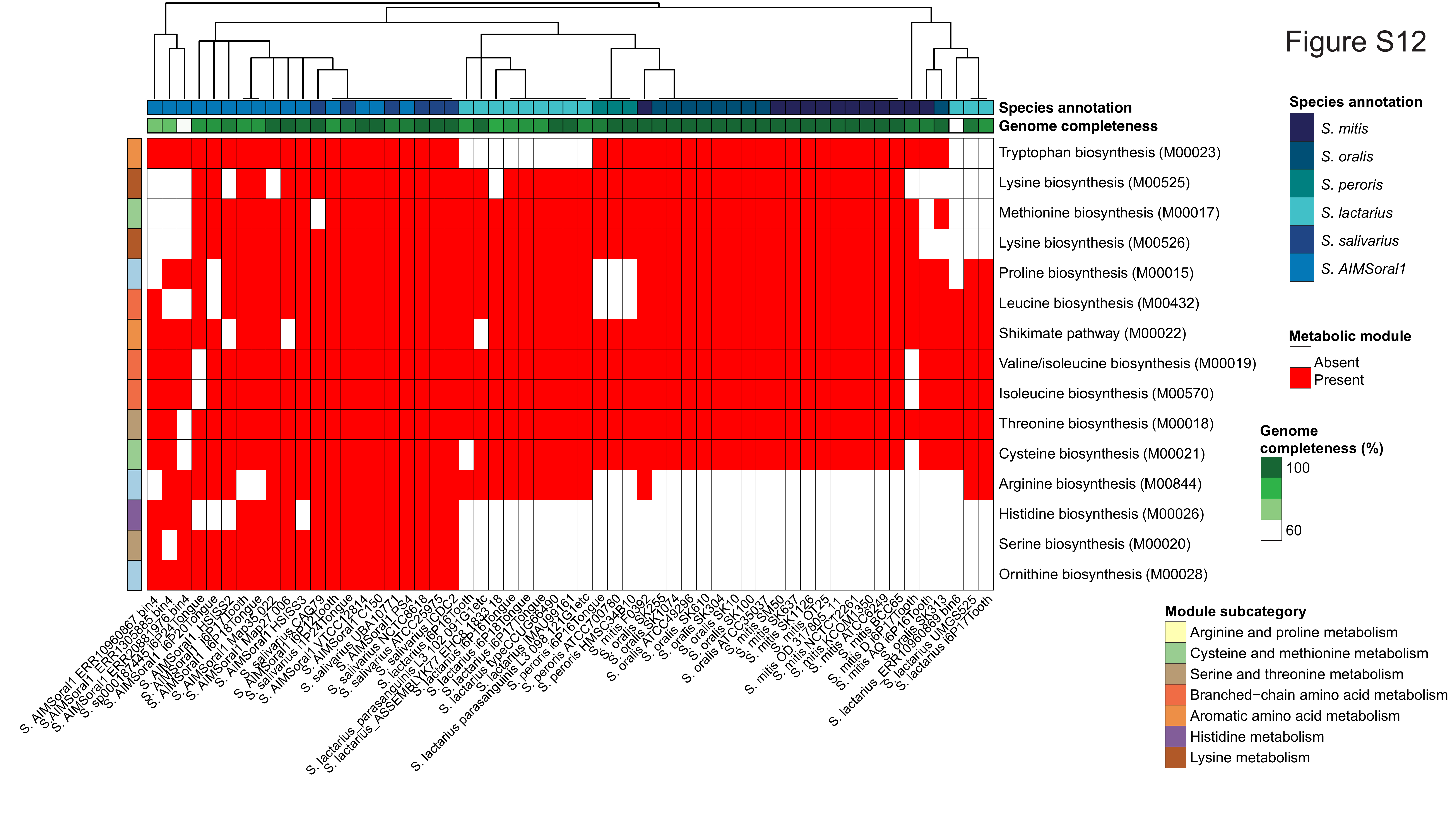

Supplement: S12 Fig — Presence/absence heatmap of 15 amino acid biosynthesis modules (KEGG) across 57 infant-associated Streptococcus genomes from six species. Red indicates module presence; white indicates absence. Hierarchical clustering (top dendrogram) groups genomes by metabolic similarity, with species identity and genome completeness shown by color. Streptococcus AIMSoral1 and S. salivarius uniquely retain arginine, histidine, serine, and ornithine biosynthesis pathways that are absent in S. mitis, S. oralis, S. peroris and S. lactarius. (TIF) [file pcbi.1013185.s012.tif]

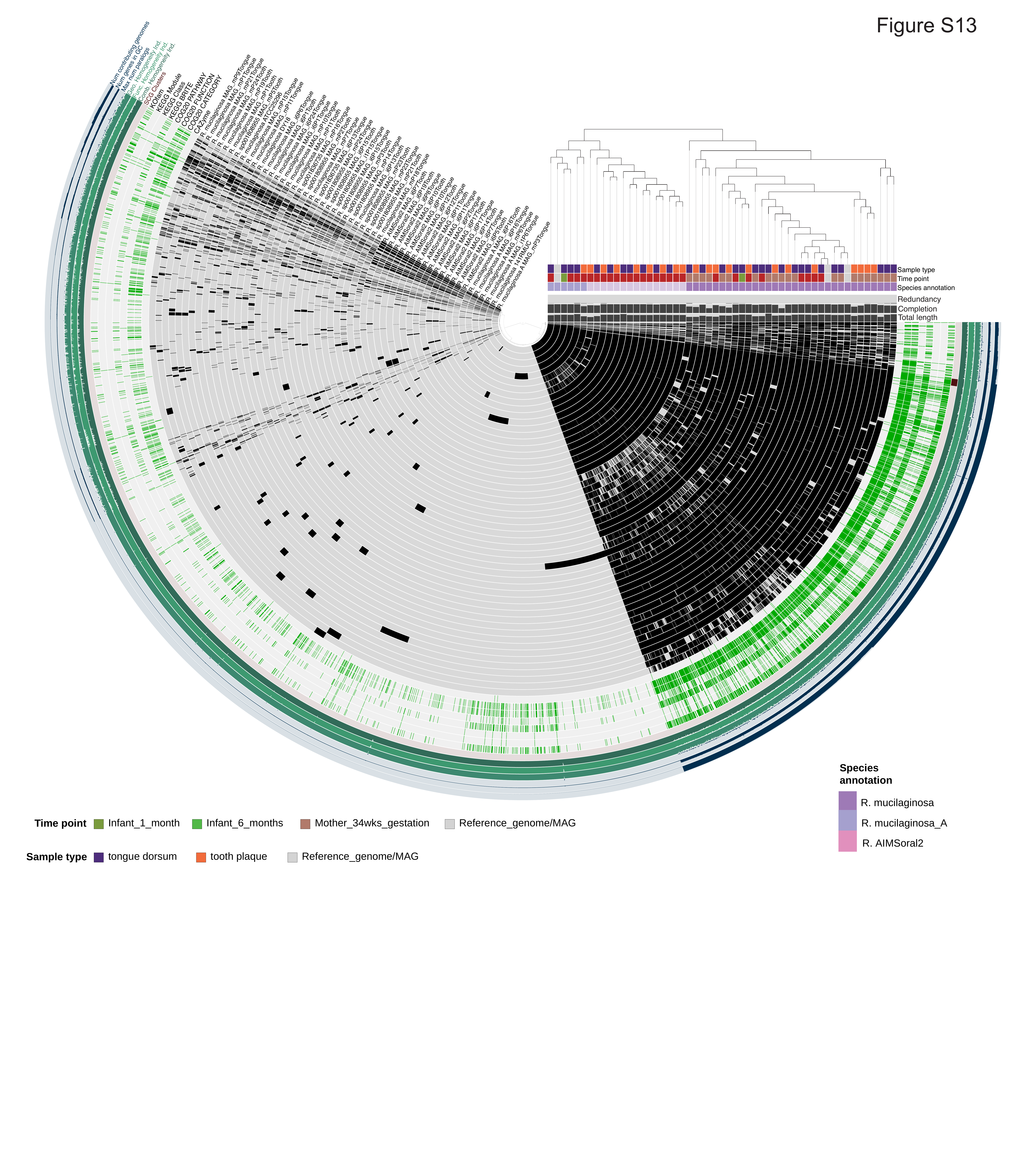

Supplement: S13 Fig — The figure shows the pangenomic characteristics of infant-associated Rothia species sorted by gene presence/absence. The pangenome includes MAGs reconstructed from AIMS oral samples (sampling location: tongue dorsum = purple, tooth plaque = orange) collected at different timepoints (mother 34wks gestation = brown, infant 1 month = green, infant 6 months = red). Reference genomes are given in grey. Species annotations as well as bars showing genome/MAG total length, completion and redundancy are shown for each strain. For each gene cluster, metrics such as number of genomes where a gene cluster is present (’num of contributing genomes) and various functional annotations are shown. (TIF) [file pcbi.1013185.s013.tif]

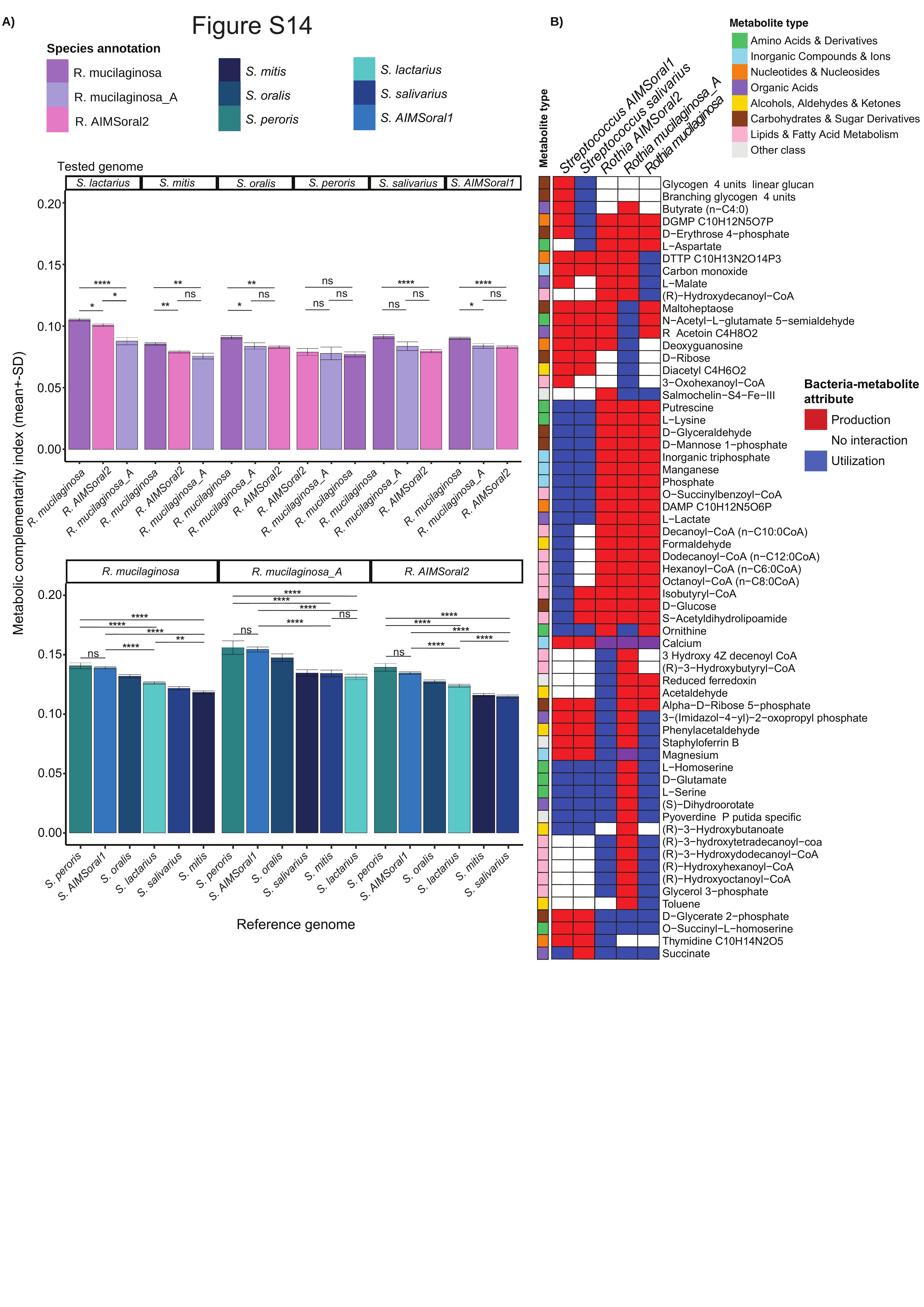

Supplement: S14 Fig — A) Bargraphs show the average metabolic complementarity index (±SD) for pairwise interactions among infant-associated Streptococcus and Rothia, and vice versa. For each complementarity species pair, the reference species is shown on the x-axis, while the other species is shown in the facet on top of the bar. B) Heatmap showing bacterial-metabolite bipartite interactions production = red, utilization = blue, both=purple, no interaction = white) potentially occurring among Streptococcus AIMSoral1, Rothia AIMSoral2 and their closely related infant-associated species in our study. Metabolites are classified by their metabolite type. (TIF) [file pcbi.1013185.s014.tif]

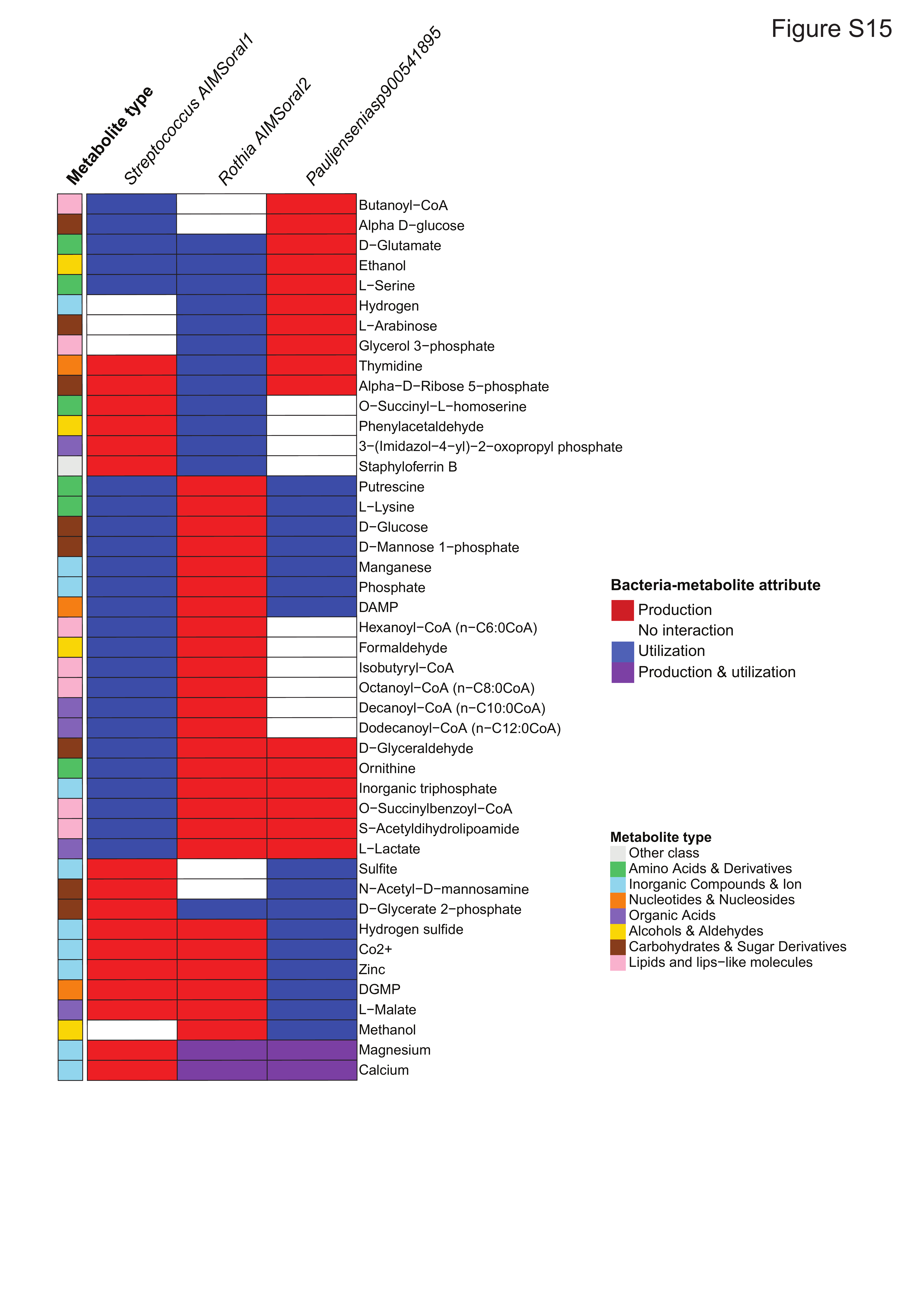

Supplement: S15 Fig — Heatmap showing unfiltered PhyloMInt PTM output of bacterial-metabolite bipartite interactions (production = red, utilization = blue, both=purple, no interaction = white) potentially occurring among species in the tongue network. Metabolites are classified by their metabolite type. Also intracellular CoA and phosphate derivatives, unlikely to be transferred, are included in the heatmap. (TIF) [file pcbi.1013185.s015.tif]

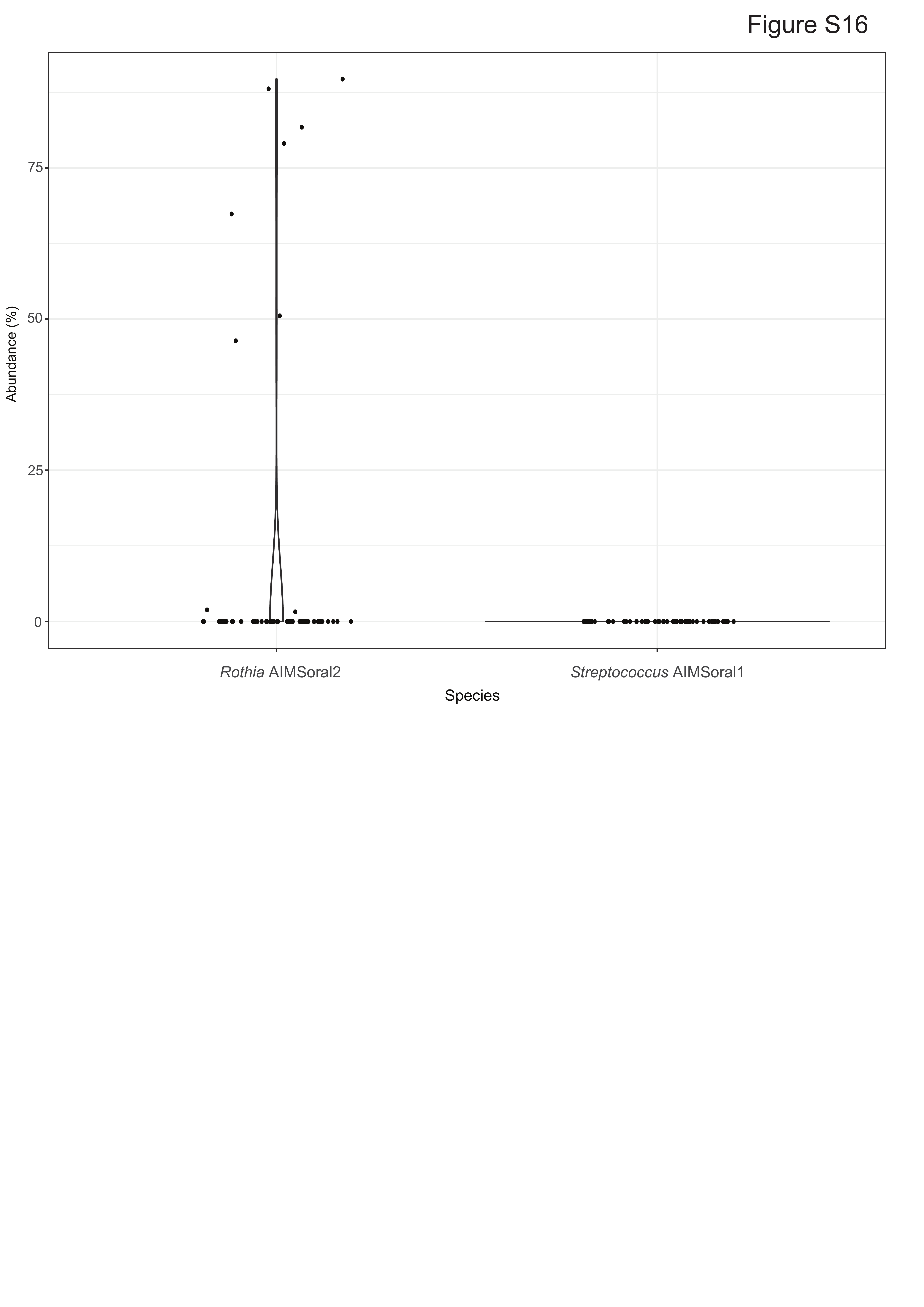

Supplement: S16 Fig — (TIF) [file pcbi.1013185.s016.tif]

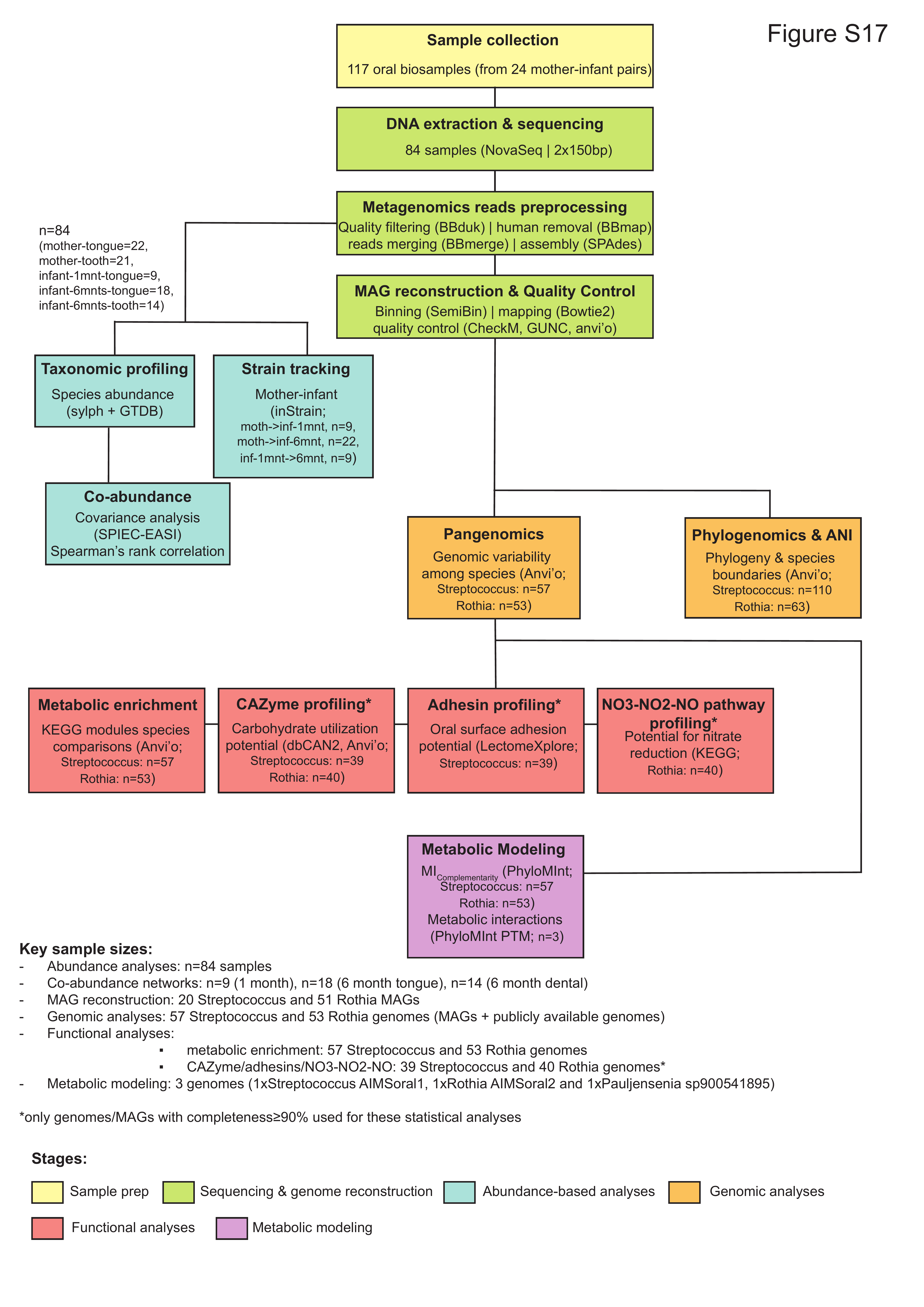

Supplement: S17 Fig — Comprehensive flowchart showing the analytical pipeline from sample collection through final analyses. The workflow is organized into six color-coded stages: sample preparation, sequencing and genome reconstruction, abundance-based analyses, genomic analyses, functional analyses and metabolic modeling. Beginning with 117 oral biosamples from 24 mother-infant pairs, the pipeline includes metagenomic sequencing, MAG reconstruction and quality control, followed by taxonomic profiling, co-abundance analysis, strain tracking, comparative genomics, functional characterization and metabolic modeling. Sample sizes for each analytical step are detailed in each box and at the bottom of the figure. (TIF) [file pcbi.1013185.s017.tif]

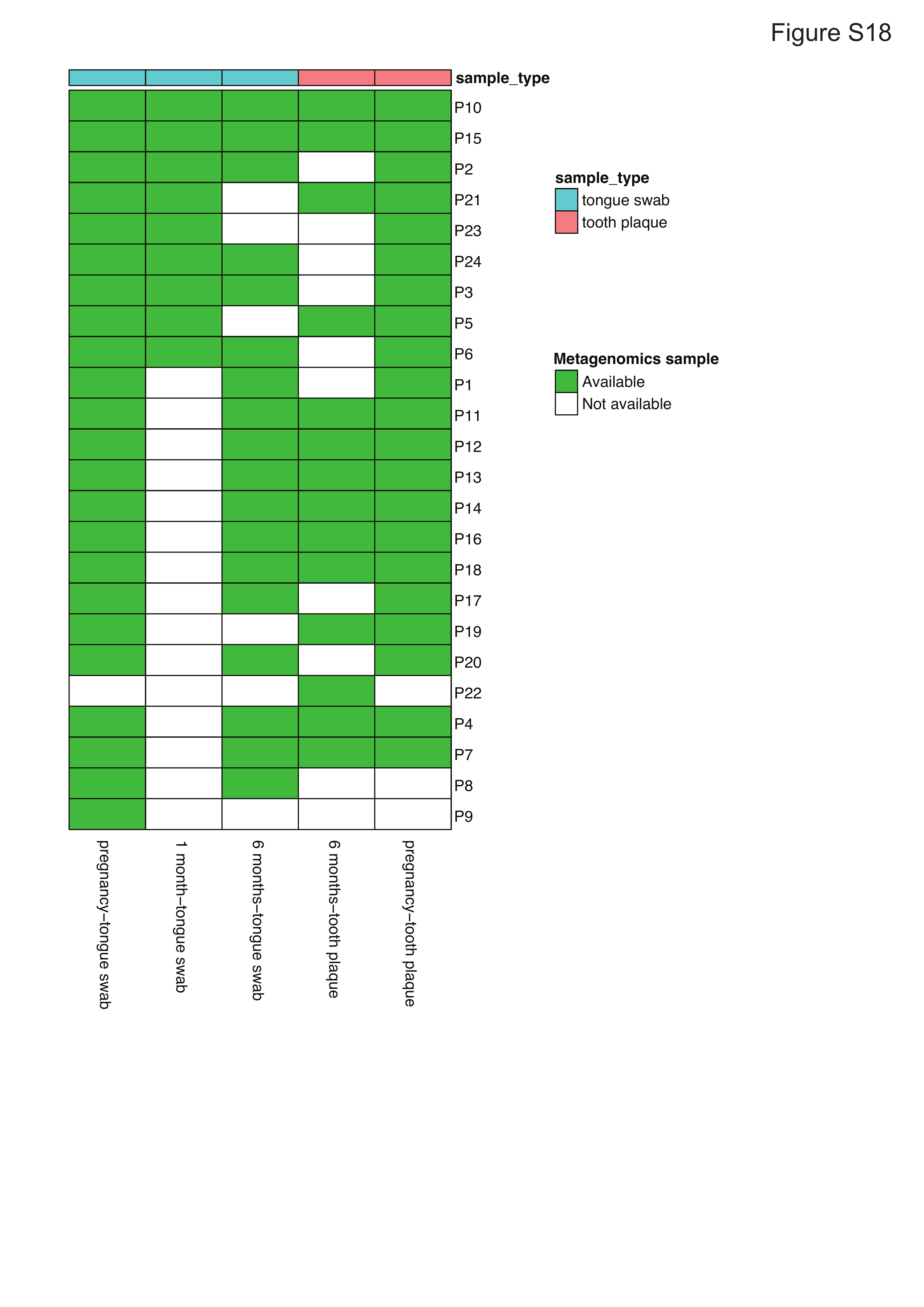

Supplement: S18 Fig — Heatmap showing sample availability for the 24 mother-infant pairs enrolled in the AIMS cohort. Rows represent individual participants (P1-P24) and columns represent sample collection timepoints and oral sites. Green: sample available for sequencing; white: sample not available. Cyan: tongue swab; pink: tooth plaque. Maternal samples were collected during pregnancy (third trimester), and infant samples were collected at 1 month (tongue only; no erupted teeth) and 6 months (tongue and tooth when dentition present). (TIF) [file pcbi.1013185.s018.tif]
