# Supplementary material for: Unique ecology of co-occurring functionally and phylogenetically undescribed species in the infant oral microbiome
Source: PLoS Comput Biol. 2026 Mar 5;22(3):e1013185. doi: 10.1371/journal.pcbi.1013185 (PMC13004514; doi:10.1371/journal.pcbi.1013185)
Supplement: Attachment — Submitted filename: Reviewer_Comments_06172025.pdf [file pcbi.1013185.s037.pdf]

1. It would strengthen the manuscript to expand the Introduction by clarifying the purpose/rationale and relevance of the network analysis. Currently, the rationale for incorporating co-occurrence networks and how they contribute to the interpretation of microbial interactions is somewhat lacking. Providing additional information would help justify this analytical approach and enhance the overall objective of the study.
2. The manuscript presents several layers of data pre-processing and quality control steps, but these are currently spread across many short subsections, which may reduce readability. For instance, sections from "Pre-processing of metagenomic reads" to "Metagenomic species profiling and strain tracking" could be considered as part of a unified pre-processing workflow. To improve clarity, I suggest consolidating these into a single subsection or grouping them under a "Pre-processing" section. Additionally, I strongly recommend including a flowchart or schema figure summarizing the step-by-step data processing pipeline. This would greatly enhance the transparency and reproducibility of the study, as is commonly done in bioinformatics studies.
3. Since the network is inferred based on abundance or taxa count data, it would be more accurate to describe it as a co-abundance network rather than a co-occurrence network. I suggest clarifying this point in the Methods section and also reflecting this terminology when presenting the rationale for the network analysis in Introduction section
4. The current network analysis is based on Spearman's rank correlations, which represent marginal (unadjusted) pairwise associations and do not account for the presence of other taxa when estimating co-abundance relationships. This is analogous to interpreting univariable regression results in the presence of multiple covariates. Given the compositional and interdependent nature of microbial communities, I strongly encourage the authors to consider whether partial correlation-based approaches or more advanced network inference methods would be more appropriate. Established alternatives such as SPIEC-EASI (graphical model-based) or regression-based methods like SOHPIE can better account for indirect effects and improve the specificity of inferred interactions.
5. As noted earlier, co-occurrence or species interaction patterns cannot be fully captured using marginal (unadjusted) correlation-based methods. The authors state that "pairwise co-occurrence patterns across ages" were inferred using hierarchical clustering and Spearman's rank correlations. However, this approach does not account for the compositional nature of microbiome data, nor does it adjust for potential confounders such as age or interactions with other taxa. As a result, the strength of connections (edges) in the resulting network may reflect spurious

associations driven by shared abundance patterns or unmeasured covariates. I encourage the authors to consider network inference methods that can incorporate covariate adjustment or control for other taxa, to more robustly infer species interactions.

6. The manuscript states that no metabolic module was exclusively enriched in Streptococcus AIMSoral1, yet a detailed discussion follows regarding its distinct amino acid metabolic profile. While this section is informative, the interpretation may come across as stronger than what the data directly support. It would be helpful either to reframe this discussion more cautiously or to include differential abundance analyses to substantiate the claim.
7. The manuscript uses MIcomplementarity to infer species interactions, but it is unclear how this metric should be interpreted in a biological or statistical sense. It would be helpful to clarify what the scores represent, whether they have a defined scale or units, and how they were validated. In particular, do these scores correspond to any known or experimentally supported interspecies metabolic interactions?
8. The identification of CAZymes such as GH66 and GT11 adds genomic depth, but the downstream functional interpretations like mucosal colonization or EPS degradation seem speculative in the absence of expression data or functional assays. It may strengthen the manuscript to either soften these interpretations or clarify the limitations if you agree.
9. Most results are presented at the species level, which is a common approach in metagenomic studies. However, given that genome-resolved analysis allows for finer resolution, it may be worth clarifying whether strain-level differences were considered or whether species-level resolution was sufficient for the study's objectives.
10. The manuscript includes metabolic enrichment analysis as a downstream step, but its role within the overall study framework is not clearly articulated. A brief explanation of the rationale for including this analysis and how it relates to or complements the co-abundance network approach would help clarify what additional biological insights it offers beyond the network analysis.
11. In the Results section, several additional analyses are presented, including phylogenomic, pangenome, and enrichment analyses. These appear to serve as supplementary layers to the main objective of the study, which focuses on co-abundance network analysis. For clarity and improved readability, it may be helpful to group these components into a dedicated subsection or a separate section titled something like "Additional Downstream Analyses."

12. In this study, authors primarily focused on the most prevalent species. Have the authors examined whether similar or substantially different patterns emerge when considering less abundant species that are not overly sparse and have been highlighted in recent clinical studies for their biological relevance? Including this as a sensitivity analysis could help assess the robustness of the findings and offer additional insights into underrepresented taxa.
