# Supplementary material for: Unique ecology of co-occurring functionally and phylogenetically undescribed species in the infant oral microbiome
Source: PLoS Comput Biol. 2026 Mar 5;22(3):e1013185. doi: 10.1371/journal.pcbi.1013185 (PMC13004514; doi:10.1371/journal.pcbi.1013185)
Supplement: Attachment — Submitted filename: PLoS_CB reviews.docx [file pcbi.1013185.s039.docx]

**Editorial and reviewer comments in black, author responses in green**

Thank you for submitting your manuscript to PLOS Computational Biology. After careful consideration, we feel that it has merit but does not fully meet PLOS Computational Biology's publication criteria as it currently stands. Therefore, we invite you to submit a revised version of the manuscript that addresses the points raised during the review process.

**Authors:** We thank the editors for the opportunity to revise our manuscript. We have now addressed all editorial and reviewer comments.

Please include the following items when submitting your revised manuscript:

* A rebuttal letter that responds to each point raised by the editor and reviewer(s). You should upload this letter as a separate file labeled 'Response to Reviewers'. This file does not need to include responses to formatting updates and technical items listed in the 'Journal Requirements' section below.

* A marked-up copy of your manuscript that highlights changes made to the original version. You should upload this as a separate file labeled 'Revised Manuscript with Track Changes'.

* An unmarked version of your revised paper without tracked changes. You should upload this as a separate file labeled 'Manuscript'.

**Authors:** We have included all three required documents and have updated the main text and supplemental figures according to the guidelines..

**Reviewers' comments:**

Reviewer's Responses to Questions

Comments to the Authors:
Please note here if the review is uploaded as an attachment.

**Authors:** Reviewer #1 provided the same comments in the text and as a separate file, we have answered them in-line below.

**Reviewer #1:** Below are my comments and I have uploaded it as a separate document as well.

1. It would strengthen the manuscript to expand the Introduction by clarifying the purpose/rationale and relevance of the network analysis. Currently, the rationale for incorporating co-occurrence networks and how they contribute to the interpretation of microbial interactions is somewhat lacking. Providing additional information would help justify this analytical approach and enhance the overall objective of the study.

**Authors:** *We thank the reviewer for this suggestion. We have expanded the Introduction (L72-73) to better clarify the rationale for network analysis. The revised text now explicitly explains that interaction network structure determines community assembly trajectories and ecological stability and emphasizes why understanding community-wide interaction patterns is essential for predicting how early colonization events shape long-term microbiome development. This additional context better justifies our network-based analytical approach and its contribution to understanding microbial interactions in the infant oral cavity.*

2. The manuscript presents several layers of data pre-processing and quality control steps, but these are currently spread across many short subsections, which may reduce readability. For instance, sections from “Pre-processing of metagenomic reads” to “Metagenomic species profiling and strain tracking” could be considered as part of a unified pre-processing workflow. To improve clarity, I suggest consolidating these into a single subsection or grouping them under a “Pre-processing” section. Additionally, I strongly recommend including a flowchart or schema figure summarizing the step-by-step data processing pipeline. This would greatly enhance the transparency and reproducibility of the study, as is commonly done in bioinformatics studies.

**Authors:** *We agree with the reviewer that consolidating related methodological sections improves readability. We have merged the "DNA extraction and sequencing", "Pre-processing of metagenomic reads" and "MAG reconstruction" sections into a unified "Metagenomic sequencing and genome reconstruction" section. However, we have kept other sections (such as "Metagenomic species profiling and strain tracking" "Phylogenomic and genetic relatedness analyses" and downstream analytical sections) as separate subsections, as these represent distinct analytical workflows with different purposes and further consolidation would reduce clarity. Additionally, we have added a flowchart (S17 Fig) summarizing the step-by-step data processing pipeline from sample collection through genome reconstruction and downstream analyses to enhance the transparency and reproducibility of our bioinformatics workflow.*

3. Since the network is inferred based on abundance or taxa count data, it would be more accurate to describe it as a co-abundance network rather than a co-occurrence network. I suggest clarifying this point in the Methods section and also reflecting this terminology when presenting the rationale for the network analysis in Introduction section.

**Authors:** *We thank the reviewer for this terminological clarification. We originally used "co-occurrence" as a broader term that is commonly used in microbial ecology literature, but we agree that "co-abundance" is more technically accurate, given that our network is specifically based on abundance correlations rather than presence/absence patterns. We have revised the terminology throughout the manuscript to consistently use "co-abundance network" instead of "co-occurrence network."*

4. The current network analysis is based on Spearman’s rank correlations, which represent marginal (unadjusted) pairwise associations and do not account for the presence of other taxa when estimating co-abundance relationships. This is analogous to interpreting univariable regression results in the presence of multiple covariates. Given the compositional and interdependent nature of microbial communities, I strongly encourage the authors to consider whether partial correlation-based approaches or more advanced network inference methods would be more appropriate. Established alternatives such as SPIEC-EASI (graphical model-based) or regression-based methods like SOHPIE can better account for indirect effects and improve the specificity of inferred interactions.

**Authors:** *We agree with the reviewer that using the SPIEC-EASI is a more robust approach than using Spearman's rank correlation correlations. We have adjusted our analysis and manuscript accordingly (see Results “Streptococcus AIMSoral1 and Rothia AIMSoral2 show significant co-abundance in infant oral ecosystems”). Initially we used spearman correlations because they are easily interpretable. We have included SPIEC-EASI results (S4 Table and S6 & S8 Figs) and computed spearman’s rank correlation (S4-5 Table and S7 Fig) for the species pairs found to be co-abundant by SPIEC-EASI to improve interpretability in* the Supplementary Information. We believe this *provides both accuracy and interpretability. In addition, we have added a detailed methods section ("Co-abundance network inference and correlation analyses") describing both analytical approaches.
We are glad to report that the new results support the previous observations regarding the central/focal species (Streptococcus AIMSoral1, Rothia AIMSoral2), with only marginal changes regarding the peripheral species. The SPIEC-EASI result indicate that only the three species Streptococcus AIMSoral1, Rothia AIMSoral2 and Pauljensenia sp900541895 (renamed from AIMSoral4 as this species was not functionally characterized like Streptococcus AIMSoral1 and Rothia AIMSoral2) form a three-species module (with significant co-abundance for all pairwise interactions) on the tongue dorsum of six-month-old infants, while the previously included Veillonella* sp018367495 *(renamed from AIMSoral3) is co-abundant with Rothia AIMSoral2 and Pauljensenia sp900541895, but not Streptococcus AIMSoral1. Hence we have focused our analysis for Figure 5 on Streptococcus AIMSoral1, Rothia AIMSoral2 and Pauljensenia sp900541895. In accordance with the new results, we have also re-named Pauljensenia sp900541895 to Pauljensenia sp900541895 in the main text, figures and supplementary information.*

5. As noted earlier, co-occurrence or species interaction patterns cannot be fully captured using marginal (unadjusted) correlation-based methods. The authors state that “pairwise co-occurrence patterns across ages” were inferred using hierarchical clustering and Spearman’s rank correlations. However, this approach does not account for the compositional nature of microbiome data, nor does it adjust for potential confounders such as age or interactions with other taxa. As a result, the strength of connections (edges) in the resulting network may reflect spurious associations driven by shared abundance patterns or unmeasured covariates. I encourage the authors to consider network inference methods that can incorporate covariate adjustment or control for other taxa, to more robustly infer species interactions.

**Authors:** *We thank the reviewer for his additional comment on this matter and believe that we have broadly answered this point in our response to the previous comment (4.) by changing the network inference method to SPIEC-EASI. Probably due to the limited number of available samples the SPIEC-EASI results showed a number of taxa that only occur in one sample to be co-abundant, prompting us to perform a sensitivity analysis (L574-576; S5 Fig & S3 Table), which indicated that including species present in at least 5 samples resulted in a robust network.
To address these changes, we have adjusted the mentioned sentence to reflect our improved approach:”*To elucidate potential species interactions, we computed a co-abundance network of species present in at least 5 samples (as indicated by a sensitivity analysis, see Methods) using SPIEC-EASI for tongue and dental biofilm samples, separately (S5-6 Fig, S3-4 Tables)”*. We corroborated these associations using Spearman's rank correlations and retained only significant relationships.”*

6. The manuscript states that no metabolic module was exclusively enriched in Streptococcus AIMSoral1, yet a detailed discussion follows regarding its distinct amino acid metabolic profile. While this section is informative, the interpretation may come across as stronger than what the data directly support. It would be helpful either to reframe this discussion more cautiously or to include differential abundance analyses to substantiate the claim.

**Authors:** *We thank the reviewer for this observation. We have reformulated our discussion to make sure that our interpretations do not overstated the uniqueness of amino acid metabolism as a distinguishing feature of Streptococcus AIMSoral1. We have addressed this concern through two complementary approaches:*

1. *We have created a heatmap (S12 Fig; referred to at L275-276) showing the distribution of all amino acid biosynthesis pathway modules across infant-associated Streptococcus genomes. This figure illustrates that while Streptococcus AIMSoral1 and S. salivarius both possess more complete amino acid biosynthesis pathways compared to other infant-associated species, these capabilities are shared between the two species and are not exclusive to Streptococcus AIMSoral1.*
2. *We have revised the discussion (L424-428) to present a more nuanced interpretation. The revised text now:*
   a. *Acknowledges that amino acid metabolism capabilities may represent important adaptations facilitating oral colonization during the weaning period*
   b. *Explicitly notes that these metabolic features are not unique to Streptococcus AIMSoral1 and therefore cannot alone explain why Streptococcus AIMSoral1 predominates in 6-month AIMS infants while S. salivarius is notably absent*
   c. *Highlights other distinguishing features (particularly the fimA adhesin present in Streptococcus AIMSoral1 but absent in S. salivarius) that may provide additional specificity for oral cavity colonization*

*We believe these revisions provide a more balanced and scientifically accurate interpretation that recognizes the potential functional importance of amino acid metabolism while appropriately acknowledging it is a shared rather than unique feature.*

7. The manuscript uses MIcomplementarity to infer species interactions, but it is unclear how this metric should be interpreted in a biological or statistical sense. It would be helpful to clarify what the scores represent, whether they have a defined scale or units, and how they were validated. In particular, do these scores correspond to any known or experimentally supported interspecies metabolic interactions?

**Authors:** *We thank the reviewer for this request for clarification. We used the definition of MIcomplementarity by Lam et al. 2020, but we agree that while the PhyloMInt tool is established the metric is not widely known. Hence, we have added a detailed explanation of MIcomplementarity to the Methods (L640-650). MIcomplementarity quantifies the potential for metabolic cross-feeding between species pairs based on genomic capabilities. It measures the proportion of essential compounds (seed compounds) required by one species that can be biosynthesized by another species' metabolic network. The metric theoretically ranges from 0 to 1, where 1 indicates complete biosynthetic complementarity (one species can provide all compounds the other requires but cannot produce) and 0 indicates no support potential. The metric is asymmetric, reflecting directional dependencies. This approach has been validated in previous studies of microbial interactions (Lam et al. 2020, PLoS Comput Biol; Peng et al. 2024, iMeta).
To further contextualize the biological relevance of the MIcomplementarity scores observed in our study, we performed an additional analysis using a positive control from the literature (L652-656). We computed MIcomplementarity for Phocaeicola dorei and Lachnoclostridium symbiosum, two species whose metabolic interactions have been experimentally validated through co-culture experiments and metabolomics (Hirmas et al. 2022, mSystems). This study demonstrated that these species interact via multiple metabolites in co-culture. Our analysis using publicly available genomes from this study yielded MIcomplementarity scores of 0.103 (P. dorei supporting L. symbiosum) and 0.154 (L. symbiosum supporting P. dorei; S12 Table; L331-336). These values are comparable to the scores we observe for key species pairs in our infant oral microbiome study, providing empirical support that the MIcomplementarity range we report is consistent with experimentally validated metabolic interactions.
Importantly, MIcomplementarity represents theoretical predictions based on genomic potential, not confirmed interactions. It provides an estimate of cross-feeding potential that requires experimental validation. To address this concern and strengthen the biological plausibility of our predictions, we have now added genomic support for key predicted exchanges by confirming the presence of transporters for a number of amino acids and metal ions (Fig 5D, S14-15Tables). However, we explicitly acknowledge in the Discussion (L481-484) that experimental validation through co-culture experiments and metabolomics is needed to confirm these predicted metabolic interactions.*

8. The identification of CAZymes such as GH66 and GT11 adds genomic depth, but the downstream functional interpretations like mucosal colonization or EPS degradation seem speculative in the absence of expression data or functional assays. It may strengthen the manuscript to either soften these interpretations or clarify the limitations if you agree.

**Authors:** *We agree with the reviewer that functional interpretations based solely on genomic data require caution in the absence of expression or functional validation. We have revised the Discussion (L434-436) to use more conservative language when discussing CAZyme functions, focusing on GH66 and GH87 (both highlighted in the Results; Fig 3) as these glycoside hydrolases are most relevant to the discussion of carbohydrate degradation in the oral environment. The revised text now: (1) reports the genomic finding of CAZyme enrichment as observed data, (2) briefly notes what these enzyme families are known to do in other systems based on literature and (3) explicitly acknowledges that whether these functions provide advantages in the infant oral cavity remains to be experimentally validated. This approach presents the genomic observations without overstating their functional implications in the specific context of infant oral colonization.*

9. Most results are presented at the species level, which is a common approach in metagenomic studies. However, given that genome-resolved analysis allows for finer resolution, it may be worth clarifying whether strain-level differences were considered or whether species-level resolution was sufficient for the study’s objectives.

**Authors:** *We thank the reviewer for this methodological consideration. While our study focuses on the identification and characterization of bacterial species, we incorporated strain-level analyses where appropriate: we performed strain-level transmission analysis using inStrain (Methods L565-566), which revealed rare strain sharing between mothers and infants (2 events; S2 Table). Additionally, our pangenomic analyses (S11 and S13 Figs) capture within-species variation by examining gene presence/absence across 14 Streptococcus AIMSoral1 and 15 Rothia AIMSoral2 MAGs. These analyses indicate minimal differences between conspecific strains of these species.
However, species-level resolution is most appropriate for our study's primary objectives: (1) identifying and characterizing novel species, (2) determining functional features distinguishing these species from phylogenetically related species, and (3) predicting between-species metabolic interactions.*

10. The manuscript includes metabolic enrichment analysis as a downstream step, but its role within the overall study framework is not clearly articulated. A brief explanation of the rationale for including this analysis and how it relates to or complements the co-abundance network approach would help clarify what additional biological insights it offers beyond the network analysis.

**Authors:** *We thank the reviewer for this suggestion. While we had included rationales for each analytical section, we have now strengthened these throughout the Results to better articulate how each analysis contributes to our overall understanding of these undescribed species' ecology. Specifically, our analytical framework comprises complementary approaches that address different biological questions: (1) identification of 2 novel species in the oral microbiome of 6-months old children; (2) co-abundance network analysis identifying which species associate with each other (L146-148); (3) species delineation through phylogenomics and average nucleotide identity (ANI; L177-180); (4) functional and (5) metabolic characterization revealing why each species is adapted to the infant oral cavity at this developmental stage by identifying functional capabilities that distinguish them from phylogenetically related species (L233-236); and (6) metabolic interaction modeling demonstrating how these co-occurring species might be metabolically linked to each other through predicted nutrient exchanges (L327-328 and L353-355). Together, these analyses provide a comprehensive picture from ecological patterns to functional adaptations to mechanistic interactions. We have clarified these distinct rationales in the Results sections where each analysis is introduced.*

11. In the Results section, several additional analyses are presented, including phylogenomic, pangenome, and enrichment analyses. These appear to serve as supplementary layers to the main objective of the study, which focuses on co-abundance network analysis. For clarity and improved readability, it may be helpful to group these components into a dedicated subsection or a separate section titled something like “Additional Downstream Analyses.”

**Authors:** *We respectfully disagree with the characterization of co-abundance network analysis as the main objective of our study. Our manuscript comprises six major analytical sections, each addressing a distinct aspect of these undescribed species' biology: (1) taxonomic identification and prevalence, (2) co-abundance patterns, (3) genomic novelty confirmation through phylogenomics, (4) functional characterization through comparative genomics, (5) metabolic capabilities, and (6) predicted metabolic interactions. The phylogenomic, pangenomic and enrichment analyses are not supplementary, they are essential for establishing that these are undescribed species and characterizing their unique functional adaptations to the infant oral cavity. These analyses are reflected in our main figures (Figures 2-4) and represent core findings of the study. To clarify this more, we have ensured that the rationale for each section is clearly stated to improve readability.*

12. In this study, authors primarily focused on the most prevalent species. Have the authors examined whether similar or substantially different patterns emerge when considering less abundant species that are not overly sparse and have been highlighted in recent clinical studies for their biological relevance? Including this as a sensitivity analysis could help assess the robustness of the findings and offer additional insights into underrepresented taxa.

**Authors:** *We appreciate the reviewer’s comment and important suggestions. We apologize for the unclear formulation in the first version of the manuscript and would like to highlight that the taxonomic microbiome characterization did not only focus on the most abundant or prevalent species (Figure 1; Section “Undescribed Streptococcus and Rothia species emerge as predominant in six-month infant oral microbiome“). As detailed in the manuscript and the title, our broad initial analysis revealed two abundant novel species with a unique ecology which we followed up throughout the manuscript. Additionally, following the re-analysis of our co-abundance network using SPIEC-EASI (see response to Comment 4), we have adopted an approach based on the reviewer’s suggestion that directly addresses this concern. Rather than restricting network inference to abundant species (we used ≥1% relative abundance in the first version of the manuscript), we now include all species detected in the infant oral microbiome, applying only a modest prevalence-based filter (present in ≥5 samples) to exclude overly sparse taxa. We determined this prevalence threshold through a sensitivity analysis, which indicated that including species present in fewer than 5 samples resulted in seemingly higher numbers of false positive connections at six months of age (S5 Fig & S3 Table). The co-abundance network (S6 Fig, S3-4 Tables) confirmed the contribution of one underrepresented species (Pauljensenia sp900541895), which was included in the metabolic interaction model (Figure 5). Since we were not able to recover MAGs of Pauljensenia sp900541895, it was not included in the genomic characterization part of the manuscript. This has been clarified in the revised manuscript* (L180-181). *Furthermore, in the tongue network, the focal species of the manuscript (Streptococcus* AIMSoral1 and *Rothia* AIMSoral2) both exhibited negative co-abundance associations with *Campylobacter concisus (*a species associated with inflammatory bowel disease, *Park et al. 2025*) *while Streptococcus* AIMSoral1 additionally showed a negative association with *Prevotella melaninogenica (*a species implicated in oral inflammation and periodontitis in adults *(Könönen et al. 2022). We have added a new supplementary figure (S8 Fig) highlighting the positive and negative co-abundance interactions of the focal species module and addressed these findings in the Methods (L582-584), Results (L164-168) and Discussion (L475-477) sections of the revised manuscript.*

**Reviewer #2:** In this work, authors performed a metagenomic analysis on infant and mother oral microbiomes.

1. In general, all analysis processed were implemented on existing tools and methods, with limited novelty on methodology, which can hardly fit the scope of PLoS CB journal.

**Authors:** *While we respect the reviewer's opinion in this matter, we chose PLoS Computational Biology as we believe it would be a great fit for this study after an assessment of the guidelines and recent publications. The insights obtained through our study represent significant biological discoveries that advance our understanding of oral microbiome assembly during infancy, which aligns with PLoS Computational Biology's scope.*

2. The study highlights a shift in microbial composition from 1 to 6 months, but the drivers of this shift (e.g., dentition, dietary transitions, maternal transmission) remain underexplored. Longitudinal analyses with more frequent sampling points could clarify how developmental milestones influence the emergence of the novel species.

**Authors:** *We thank the reviewer for this comment. While we agree that more fine-grained sampling could have been beneficial retrospectively, the ecology of the focal species of this study (Streptococcus AIMSoral1 and Rothia AIMSoral2) was unknown before this study. Nevertheless, we did investigate maternal transmission utilizing multiple methods, showing the rare occurrence of shared strains between maternal and infant samples. As this has been demonstrated in a recent study (Valles-Colomer et al. 2022), we merely acknowledge and report our congruent results.
We deliberately limited our interpretation regarding the drivers of compositional shifts because dentition and dietary transitions both occur within the 1-to-6-month window in our cohort (as indicated by the reviewer). As dentition and dietary transition actually completely overlap, additional intermediate sampling points would have needed to be very frequent and detailed (e.g. at least biweekly sampling with additional questionnaires dedicated to nutrition and dentition) to disentangle their individual contributions. Such samples are not available for this study, and we acknowledge the limitations of the study in our discussion (L413-414). Nevertheless, this does not limit the validity of the study and we want to emphasize that the primary focus of our manuscript, as reflected in the title and scope, is the characterization of two previously undescribed, highly abundant species at six months and their functional ecology within the developing oral microbiome.*

3. Furthermore, while strain sharing between mothers and infants is noted as rare, investigating vertical transmission of functional traits (rather than just strains) may reveal hidden maternal influences.

**Authors:** *Strain-level vertical transmission is a well-established concept in human microbiome research, particularly for parent-child transmission (refs: Korpela et al. 2018; Ferretti et al. 2018; Yassour et al. 2018; Brito et al. 2019; Valles-Colomer et al. 2022), and our strain tracking approach follows these established methods. To better understand the reviewer request, we investigated the literature on the topic of "vertical transmission of functional traits", yet we could not find any publication that would indicate that posted a concept which could help us to “reveal hidden maternal influences”. Most literature in this area was focussed on functional traits that promote person-to-person (or e.g. plant-to-plant) transmission. Another related area is concerned with functional traits that are related / required for the colonization of certain environments. Obviously, there are genetic and environmental links between mother and child that could potentially shape the infant microbiome including the potential to promote or impede the growth of microbes with specific functional traits. Such an investigation would require specific data, including detailed descriptions of the (home) environment as well maternal and infant genomic data, which is not available for this study nor for any study that we are aware of.*

*As the transmission of functional traits was not a focus of this study and requires specific additional data, answering this particular question is outside of the scope of this study.*

4. While this is a computational study, the paper would benefit from acknowledging the lack of experimental validation (e.g., co-culture, metabolomics) and explicitly outlining which predictions could be tested in future studies.

**Authors:** *We agree with the reviewer and have strengthened our acknowledgment of these limitations. We have now expanded this section to more explicitly outline testable predictions (L481-484).*

5. The metabolic modeling approach (GEMS + PhyloMInt) supports potential nutrient exchanges between co-occurring species. However, the conclusions at times read as causal. Please rephrase key statements to clarify that these are predictions rather than confirmed interactions.

**Authors:** *We agree with the reviewer that our language should more clearly distinguish between computational predictions and confirmed interactions. We have revised the manuscript throughout to use more cautious phrasing, including terms like 'predicted,' 'potential,' and 'may contribute to' rather than definitive statements. To strengthen the biological plausibility of our metabolic predictions, we have added a genomic validation step whereby we systematically identified membrane transporter genes for predicted metabolite exchanges among the three species network (newly added Methods section, Fig 5D, S14-S15 Tables). This analysis confirmed that all three species encode the necessary transporters for key predicted amino acid and metal ion exchanges, providing additional genomic support (though not experimental confirmation), for these interactions. We have also added text in our limitations section emphasizing that experimental validation through co-culture experiments and metabolomic profiling would be required to confirm these predicted interactions (L481-484). Sadly, we are not able to perform such experiments at this point in time due to a lack of isolates as well as the unavailability of additional samples.
Additionally, following SPIEC-EASI re-analysis (see response to Reviewer #1, Comment 4), we have focused our metabolic modeling (Figure 5) on the three species module within the co-abundance network: Streptococcus AIMSoral1, Rothia AIMSoral2 and Pauljensenia sp900541895 (renamed from AIMSoral4; not functionally characterized due to a lack of MAGs).*

6. The link of source code and source data is not available.

**Authors:** *We apologize for this oversight. The code and data are now available at our GitHub repository:* [*https://github.com/nicholaspucci/infant-oral-microbiome-strep-rothia*](https://github.com/nicholaspucci/infant-oral-microbiome-strep-rothia)*. Raw sequencing reads have been deposited at the European Nucleotide Archive (ENA) under BioProject accession number PRJEB88622.*

**Reviewer #3**: In this article, Pucci et al., report novel co-occurring species and provide metapangenomics and GEMS based evidence to suggest functional interactions – of relevance to our understanding of early oral microbiome development.

This work represents a valuable addition to our understanding of human oral microbiome, particularly of its development during early infancy. The study has many strengths including its longitudinal nature, access to very valuable mother-infant dyad and tongue dorsum and dental plaque samples, as well as metagenomic sequencing – enabling the identification of reported “novel” species and their potential functional interactions. However, in my view, the manuscript does not provide sufficient details to qualify all the reported observations.

**Authors:** We thank the reviewer for their accurate summary of our manuscript and their encouraging words.

Specifically, to begin with- how many samples were infact studied longitudinally? They report 24 mother-infant dyad but seemingly only 9 were available at 1-month and 14 at 6-months? Numbers were unclear for tongue versus dental as well. It would help to have a clear figure describing the sample sizes available/used per analyses to inform the reader. This is important for statistical considerations, which were absolutely missing from all figures/ legends/ methods section of the manuscript.

**Authors:** We thank the reviewer for highlighting these important clarifications. We have created a comprehensive study flowchart (see S17 Fig) showing sample collection and processing and the specific sample sizes used for each analysis throughout the manuscript: 24 mother-infant dyads enrolled, with 84 of 117 collected samples yielding sufficient DNA for sequencing (mothers: tongue n=22, dental n=21; 1-month infants: tongue n=9; 6-month infants: tongue n=18, dental n=14). Not all infants had erupted teeth at 6 months (dentition, n=21 out of 24), and dental plaque collection was not possible at 1 month. To address the reviewer's concern about sample sizes, we have created a sample availability heatmap (S18 Fig) and added sample sizes to all figure legends. Specifically: Figure 1 legend now includes sample sizes for each age/site group, while Figures 3 and 4 legends report the number of genomes used in comparative analyses. Additionally, we have expanded the "Data analysis and statistics" Methods subsection (L664) describing all statistical tests (Kruskal-Wallis with Dunn's post-hoc tests, Wilcoxon tests, Spearman’s rank correlations), updating all figure legends with statistical tests used and adding significance indicators (*, **, ***, ****,ns) directly on Figures 3 and 4.

How were the relative abundances/ prevalences defined? Lines 188-189: authors say they focussed on the functional characterization of Strept oral 1 and Rothia oral2 but not Veionella Oral 3 and Pauljensenia Oral 4 because of high abundance and prevalence but how was that determined? And where are these data? Is it possible that lower sample sizes may have affected abundances/prevalences?

**Authors:** We thank the reviewer for this important clarification request and apologize for the unclear formulation in the first version of the manuscript. In essence, we focused our functional characterization on Streptococcus AIMSoral1 and Rothia AIMSoral2 because of their undescribed taxonomic status (see phylogenomic analyses, Fig 2), their strong co-abundance patterns in the infant oral microbiome (see network analyses, S6 Fig & S3 Table), the availability of MAGs, and their prominent abundance and prevalence in our samples of 6 months old children (see Fig 1, S2 Fig).
We had planned to characterize the functional repertoire of all species within the co-abundance module initially comprising Streptococcus AIMSoral1, Rothia AIMSoral2, Veillonella sp018367495 (AIMSoral3), and Pauljensenia sp900541895 (AIMSoral4). To this end, we required the availability of MAGs (medium- and high-quality) and we successfully recovered 5 Streptococcus AIMSoral1 and 15 Rothia AIMSoral2 MAGs from our samples, enabling pangenome, comparative genomic and functional analyses (S17 Fig). In contrast, no MAGs of high enough quality were retrieved for Veillonella sp018367495 (AIMSoral3) or Pauljensenia sp900541895 (AIMSoral4) from our samples, precluding the same depth of functional characterization.
For completeness, relative abundances were calculated from Sylph profiling results (see Methods: Metagenomic species-profiling and strain tracking) and prevalence was defined as the percentage of samples in which a species was detected. These data are shown in Figure 1 and Supplementary Figure S2, which presents abundance-prevalence plots for 1-month and 6-month infant oral samples.
We apologize for the incomplete formulation for the selection procedure in the initial manuscript version. To improve clarity and address the reviewer's concern, we have revised the manuscript to more explicitly state the rationale for focusing functional characterization on Streptococcus AIMSoral1 and Rothia AIMSoral2 (L171-173) and to clarify why Pauljensenia sp900541895 was not subjected to the same depth of functional analyses. Veillonella sp018367495 (AIMSoral3) was not part of the co-abundance module and was not included in any follow up analyses in the revised version of the manuscript.
As a larger sample size would have potentially aided in the recovery of MAGs of Pauljensenia sp900541895, we have added this as an additional limitation to the discussion (L488-490).

Similarly, functional enrichments were hard to follow statistically. Was each band significantly different (e.g., Fig 3 and 4). It seemed that most species shown in Fig 3 had the potential for amino acid metabolism and fatty acid biosynthetic potential was not exclusive to Rothia oral 2 as part of Fig 4.

**Authors:** We thank the reviewer for pointing out these improvements to Figures 3 and 4. To provide additional statistical clarity, we have added significance indicators (asterisks) directly to the heatmaps in Figures 3 and 4, showing the degree of significance for each enriched feature. The asterisks represent the strongest significance level when comparing the focal species (*Streptococcus* AIMSoral1 or *Rothia* AIMSoral2) to all other species within the genus. All statistics were performed on normalized gene copy-number abundances (normalized by genome completeness) using only high-quality genomes (≥90% completeness) to ensure robust comparisons (Kruskal-Wallis with post-hoc Dunn's test, FDR-corrected). We have updated Supplementary Table S10 (now S9) to include statistics for metabolic categories and to reflect these quality filtering criteria, with detailed statistical methods provided in the figure legends.
The reviewer correctly notes that amino acid metabolism is not *exclusive* to *Streptococcus* AIMSoral1. We have created a comprehensive visualization of all amino acid biosynthesis modules across infant-associated Streptococcus species (S12 Fig), which confirms that while most species possess basic amino acid capabilities, specific pathways show distinct patterns. For example, complete arginine biosynthesis from glutamate is present only in *Streptococcus* AIMSoral1 and *S. salivarius*, as our text already acknowledges. We have revised the manuscript to consistently distinguish between significant enrichment and exclusive presence, emphasizing that it is the significantly higher copy number of these metabolic functions in Streptococcus AIMSoral1 and Rothia AIMSoral2 that distinguishes them from related species.

Similarly, in Figure 5, the metabolite production versus utilization was not specific to Rothia Oral 2 and Strept Oral 1 but was essentially shared among the 4 species of which the other 2 were not studied functionally. The conclusions drawn are limited to the analyses undertaken, however, there is potential for further data mining or adding stronger rationale for the current conclusions.

**Authors:** We acknowledge that comprehensive functional characterization of all species within the detected co-abundance module would strengthen our conclusions. However, no MAGs were retrieved for Pauljensenia sp900541895, precluding pangenome and comparative analyses (Veillonella sp018367495 was excluded as the updated co-abundance network included a module containing only Streptococcus AIMSoral1, Rothia AIMSoral2 and Pauljensenia sp900541895, also see answers reviewer 1 comments). To clarify this, we have now clarified this limitation in the Results (L181-183).
To address the reviewer's concern of the specificity of the metabolic interactions between Streptococcus AIMSoral1 and Rothia AIMSoral2 and their suggestion for additional data mining, we systematically validated predicted metabolic exchanges by searching for corresponding membrane transporters (excluding intracellular CoA and phosphate derivatives) in *Streptococcus* AIMSoral1, *Rothia* AIMSoral2 and *Pauljensenia* *sp900541895* (L656-662). We queried KEGG transporter annotations across these representative genomes for each species using an independently assembled MAG from the infant human gut of *Pauljensenia* *sp900541895* (S14-15 Tables). The analysis revealed a strong concordance between predicted exchanges and transporter presence for critical amino acids: D-glutamate, L-serine, L-lysine and ornithine (Fig5D and S15 Table; L393-399). These potential interactions, though not exclusive, represent key metabolic relationships within the three-species network given the ecological dominance of Streptococcus AIMSoral1 and Rothia AIMSoral2. Notably, *Streptococcus* possesses the complete metabolic module for arginine biosynthesis from ornithine, further supporting this predicted exchange.
We removed L-malate analysis from the revised manuscript after reanalysis showed both *Rothia* and *Streptococcus* can produce malate, eliminating the cross-feeding dependency. We now focus exclusively on metabolites with clear complementarity, strengthening our conclusions.

The manuscript will also benefit from further details in most figures and legends, with better referencing to the figures and their respective legends. The reported observations will also benefit from adding analyses at 9 month or 12 months of age, if at all those data are available. Or else from a description of why that may or may not be the case. Additionally, the limitations of the current work must be considered.

**Authors:** We have carefully reviewed all figures and their legends to ensure they provide sufficient detail for interpretation. We have enhanced figure legends with additional methodological details where appropriate and improved cross-referencing between the main text and figures throughout the Results section.
We appreciate the suggestion to include 9- or 12-month data. While the AIMS cohort includes additional timepoints, infant oral samples are currently only available at 1 and 6 months postpartum. Future analyses incorporating later timepoints would provide valuable insights into continued oral microbiome development.

Have the authors made all data and (if applicable) computational code underlying the findings in their manuscript fully available?
The [PLOS Data policy](https://journals.plos.org/ploscompbiol/s/materials-and-software-sharing) requires authors to make all data and code underlying the findings described in their manuscript fully available without restriction, with rare exception (please refer to the Data Availability Statement in the manuscript PDF file). The data and code should be provided as part of the manuscript or its supporting information, or deposited to a public repository. For example, in addition to summary statistics, the data points behind means, medians and variance measures should be available. If there are restrictions on publicly sharing data or code —e.g. participant privacy or use of data from a third party—those must be specified.

Reviewer #1: No: Full access to the data requires a data use agreement. The authors have noted the source and provided the hyperlink to the database.

Reviewer #2: No:

**Authors:** *We apologize for this oversight. The code and data are now available at our GitHub repository:* [*https://github.com/nicholaspucci/infant-oral-microbiome-strep-rothia*](https://github.com/nicholaspucci/infant-oral-microbiome-strep-rothia)*. Raw sequencing reads have been deposited at the European Nucleotide Archive (ENA) under BioProject accession number PRJEB88622.*

**References**

Brito, I. L., Gurry, T., Zhao, S., Huang, K., Young, S. K., Shea, T. P., Naisilisili, W., Jenkins, A. P., Jupiter, S. D., Castillo, D., Boussina, A., Wickremesinghe, D., Walters, W. A., Karaoz, U., Knight, R., Oliveira, R. A., Lewis, J. D., Bushman, F. D., & Alm, E. J. (2019). Transmission of human-associated microbiota along family and social networks. *Nature Microbiology*, *4*(6), 964–971. <https://doi.org/10.1038/s41564-019-0409-6>

Ferretti, P., Pasolli, E., Tett, A., Asnicar, F., Gorfer, V., Fedi, S., Armanini, F., Truong, D. T., Manara, S., Zolfo, M., Beghini, F., Bertorelli, R., De Sanctis, V., Bariletti, I., Canto, R., Clementi, R., Cologna, M., Crifò, T., Cusumano, G., ... Segata, N. (2018). Mother-to-infant microbial transmission from different body sites shapes the developing infant gut microbiome. *Cell Host & Microbe*, *24*(1), 133–145.e5. <https://doi.org/10.1016/j.chom.2018.06.005>

Hirmas, B., Gasaly, N., Orellana, G., Vega-Sagardía, M., Saa, P., Gotteland, M., & Garrido, D. (2022). Metabolic modeling and bidirectional culturing of two gut microbes reveal cross-feeding interactions and protective effects on intestinal cells. *mSystems*, *7*(6), e0064622. <https://doi.org/10.1128/msystems.00646-22>

Jo, R., Yama, K., Aita, Y., Tsutsumi, K., Ishihara, C., Maruyama, M., Takahashi, T., Ito, K., Aoki, H., Kagami, H., & Morishima, S. (2021). Comparison of oral microbiome profiles in 18-month-old infants and their parents. *Scientific Reports*, *11*(1), 861. <https://doi.org/10.1038/s41598-020-79751-8>

Könönen, E., Fteita, D., Gursoy, U. K., & Gursoy, M. (2022). Prevotella species as oral residents and infectious agents with potential impact on systemic conditions. *Journal of Oral Microbiology*, *14*(1), 2079814. <https://doi.org/10.1080/20002297.2022.2079814>

Korpela, K., Costea, P., Coelho, L. P., Kandels-Lewis, S., Willemsen, G., Boomsma, D. I., Segata, N., & Bork, P. (2018). Selective maternal seeding and environment shape the human gut microbiome. *Genome Research*, *28*(4), 561–568. <https://doi.org/10.1101/gr.233940.117>

Lam, T. J., Stamboulian, M., Han, W., & Ye, Y. (2020). Model-based and phylogenetically adjusted quantification of metabolic interaction between microbial species. *PLoS Computational Biology*, *16*(10), e1007951. <https://doi.org/10.1371/journal.pcbi.1007951>

Li, Y., Saraithong, P., Zhang, L., Dills, A., Paster, B. J., Xiao, J., Wu, T. T., & Jones, Z. (2023). Dynamics of oral microbiome acquisition in healthy infants: A pilot study. *Frontiers in Oral Health*, *4*, 1152601. <https://doi.org/10.3389/froh.2023.1152601>

Neu, A. T., Allen, E. E., & Roy, K. (2021). Defining and quantifying the core microbiome: Challenges and prospects. *Proceedings of the National Academy of Sciences*, *118*(51), e2104429118. <https://doi.org/10.1073/pnas.2104429118>

Park J-D, Lee SR, Dhennezel C, Taylor N, Dame A, Kadoki M, et al. (2025) Elucidating the role of -derived indole metabolites in gut inflammation and immune modulation. Proceedings of the National Academy of Sciences, 122(34),e2514071122. https://doi.org/10.1073/pnas.2514071122

Peng, X., Feng, K., Yang, X., He, Q., Zhao, B., Li, T., Ma, L., Wu, Y., & Ye, Y. (2024). iNAP 2.0: Harnessing metabolic complementarity in microbial network analysis. *iMeta*, *3*, e235. <https://doi.org/10.1002/imt2.235>

Sabrie, N. A. S., Halani, S., Maguire, F., Aftanas, P., Kozak, R., & Andany, N. (2023). Lachnoanaerobaculum orale bacteremia in a patient with acute myeloid leukemia and stomatitis: An emerging pathogen. *IDCases*, *33*, e01837. <https://doi.org/10.1016/j.idcr.2023.e01837>

Valles-Colomer, M., Blanco-Míguez, A., Manghi, P., Asnicar, F., Dubois, L., Golzato, D., Armanini, F., Cumbo, F., Huang, K. D., Manara, S., Masetti, G., Pinto, F., Piperni, E., Punčochář, M., Tett, A., Zolfo, M., & Segata, N. (2023). The person-to-person transmission landscape of the gut and oral microbiomes. *Nature*, *614*(7946), 125–135. <https://doi.org/10.1038/s41586-022-05620-1>

Yassour, M., Jason, E., Hogstrom, L. J., Arthur, T. D., Tripathi, S., Siljander, H., Selvenius, J., Oikarinen, S., Hyöty, H., Virtanen, S. M., Ilonen, J., Ferretti, P., Pasolli, E., Tett, A., Asnicar, F., Segata, N., Vlamakis, H., Lander, E. S., Huttenhower, C., ... Xavier, R. J. (2018). Strain-level analysis of mother-to-child bacterial transmission during the first few months of life. *Cell Host & Microbe*, *24*(1), 146–154.e4. <https://doi.org/10.1016/j.chom.2018.06.007>

Webb, K., Zain, N. M. M., Stewart, I., Fogarty, A., Nash, E. F., Whitehouse, J. L., ... & Barr, H. L. (2022). Porphyromonas pasteri and Prevotella nanceiensis in the sputum microbiota are associated with increased decline in lung function in individuals with cystic fibrosis. *Journal of medical microbiology*, *71*(2), 001481.
